# Longer leisure walking time is associated with positive self-rated health among adults and older adults: a Brazilian nationwide study

**DOI:** 10.7717/peerj.11471

**Published:** 2021-05-17

**Authors:** Diego Augusto Santos Silva

**Affiliations:** Research Center in Kinanthropometry and Human Performance, Sports Center, Universidade Federal de Santa Catarina, Florianópolis, Santa Catarina, Brazil

**Keywords:** Kinesiology, Mental health, Epidemiology, Self-Assessment, Sports

## Abstract

**Background:**

To verify the association between weekly leisure walking time and positive self-rated health in the Brazilian adult and elderly population.

**Methods:**

This cross-sectional study used information collected in 2019 across all regions of Brazil. This study included 25,785 people aged ≥ 18 years (mean = 51.6; standard deviation = 18.0) from all capitals of the Brazilian states who reported practicing walking as physical activity during leisure time. Self-rated health was the dependent variable (positive or negative). The leisure walking time/week was the main exposure and it was categorized in “150 minutes/week”, “150–299 minutes/week” and “≥ 300 minutes/week”. We used binary logistic regression to estimate odds ratio (OR) and 95% confidence intervals (95% CI) that was adjusted for relevant covariates.

**Results:**

We found that individuals who reported leisure walking for a period from 150 to 299 minutes/week and those who reported walking for a period ≥ 300 minutes/week were respectively 28% (OR = 1.28. 95% CI [1.10–1.48]) and 52% (OR = 1.52. 95% CI [1.27–1.82]) more likely of perceiving their health positively compared to those who reported walking for a period < 150 minutes/week. Individuals who reported leisure walking time <150 minutes/week had 72.3% (95% CI [70.4–74.1]) probability of perceiving their health positively. Individuals who reported leisure walking time from 150 to 299 minutes/week had 76.6% (95% CI [75.0 –78.3) probability of perceiving their health positively. On the other hand, individuals who reported leisure walking time ≥ 300 minutes/week had 79.2% probability (95% CI [77.1–81.4]) of perceiving their health positively.

**Conclusion:**

Longer leisure walking time was associated with positive self-rated health among adults and older adults in Brazil.

## Introduction

The practice of physical activity during leisure time is one of the domains of physical activity with the highest priority by public health agencies. The leisure domain stands out because it is the period when people are not linked to work activities and have time for health self-care. One of the ways to promote health self-care with is through the practice of physical activity (World Health Organization, 2007).

Among the types of physical activities that people can practice during leisure time, walking stands out for being of moderate intensity (Ainsworth et al., 2011) and, therefore, with low risk, and also for having great reach in the population, as it does not require equipment and/or spaces with high financial cost (Stamatakis et al., 2018; Celis-Morales et al., 2019; Rodriguez-Hernandez & Wadsworth, 2019). For this reason, the promotion of leisure walking is widespread worldwide, and is even the subject of public policies in many countries (Rodriguez-Hernandez & Wadsworth, 2019). Studies have shown a dose–response relationship between walking and better cardiovascular health indicators, including reduced mortality from all causes (Stamatakis et al., 2018; Celis-Morales et al., 2019). In this sense, population monitoring of this type of physical activity can guide more assertive public policies to promote physical activity.

Monitoring the practice of walking, as well as measuring the practice of physical activities, is something challenging. Measures can be self-reported, which is more usual in epidemiological studies, but they can also be objective measures (accelerometers, pedometers, etc.) (Procter-Gray et al., 2015; Reis et al., 2008; Reis et al., 2013). Different studies present different forms of measurement and different estimates of the walking prevalence during leisure time around the world (Procter-Gray et al., 2015; Reis et al., 2008; Reis et al., 2013). A study carried out in the United States of America with older adults found that one in three participants reported walking at least five days per week out of their homes; and 42% walked recreationally at least once a week (Procter-Gray et al., 2015). Research with American adults found that 49% of adults (51% of men and 47% of women) were regular walkers (e.g., regular walking was defined as walking>or = 5 d/wk,>or = 30 min/d) (Reis et al., 2008). In Brazil, Reis et al. (2013) found that 12.6% of residents of a city in the southern region reported walking during leisure time for a period ≥150 min/week”.

Measuring health indicators in the population is challenging, especially because laboratory tests, periodic medical consultations and effective health monitoring have high costs. Thus, health assessment measures of population reach are increasingly prioritized as initial screening and monitoring of the health conditions of a population. Among these measures of population reach, one of the most prominent is the self-rated health (Fayers & Sprangers, 2002; Burns et al., 2014).

Self-rated health covers not only strictly objective health aspects, such as whether or not one has any disease, but also the well-being perception, summarizing body perceptions and knowledge about the causes and consequences of diseases (Fayers & Sprangers, 2002; Höfelmann & Blank, 2007). Thus, self-rated health is related to a subjective state that has consequences for health, remaining limited to psychological constructs such as well-being, satisfaction, control over life, physical sufficiency and quality of life (Höfelmann & Blank, 2007; Burns et al., 2014).

The prevalence of positive self-rated health is also variable according to the characteristics of the investigated sample. A study developed with adults and older adults carried out in Brazil in 2013 found that the prevalence of positive self-rated health ranged from 76% to 93%, depending on the respondent’s clinical condition (Theme Filha et al., 2015). In Spain, it was found that among adults and older adults, the prevalence of positive self-rated health was 73%, but this estimate varied according to people’s health diagnosis (Malmusi et al., 2012). In Asian adults, estimates for positive self-rated health were 44.3%, 58.7%, 37.7%, and 73.7% in Bangladeshis, Indians, Nepalese, and Sri Lankans, respectively (Yaya & Bishwajit, 2020).

Different modalities of physical activities bring protective effects to various health outcomes (Mielke et al., 2020; Oja et al., 2017). Mielke et al. (2020) analyzed longitudinal information from Australian adults and found that over six years of follow-up, people who were involved in: (1) running, tennis, team sports, exercise classes and resistance training were associated with reduced odds of hypertension; (2) people involved in running, cycling, resistance training, and yoga/tai chi were associated with reduced odds of diabetes; (3) people involved in cycling, tennis, home-based exercises, resistance training, and yoga/tai chi were associated with lower odds of obesity. Oja et al. (2017) investigated participants of the Health Survey for England (HSE) and the Scottish Health Survey (SHeS) for nine years and found that significant reductions in all-cause mortality were observed due to participation in cycling, swimming, racquet sports and aerobics. No significant associations were found for participation in football and running. In addition, significant reduction in cardiovascular disease mortality was observed for participation in swimming, racquet sports and aerobics, but no significant associations were observed for cycling, running and football. Walking is considered a moderate intensity activity, but it can vary according to pace, speed and amplitude (Yates et al., 2021; Grazzi et al., 2020). Studies that have investigated the practice of walking in relation to health outcomes have reported significant reductions in the occurrence of diseases and chronic health problems with the practice of walking in populations with or without diagnosis of diseases (Ikeda et al., 2020; Tsuji et al., 2017). Furthermore, the authors of the present study believe that walking during leisure time is a less expensive form of physical activity compared to other modalities that require sophisticated spaces and materials (i.e., swimming, cycling, racquet sports) and can be a closer reality in middle- and low-income countries like Brazil and, for this reason, investigations on the practice of walking at leisure time can be useful.

A series of population studies have investigated the relationship between physical activity and self-rated health. In general, these studies demonstrated a direct relationship between level of physical activity and positive health perception in adults and older adults (Johansson et al., 2019; Cui et al., 2020; Han, 2020; Sperlich et al., 2020; Yaya & Bishwajit, 2020), and the high level of physical activity is a factor that compensated for the low socioeconomic level in the self-rated health status (Johansson et al., 2019). However, most studies have analyzed the level of general physical activity (without specifying domains) (Johansson et al., 2019; Cui et al., 2020; Han, 2020; Yaya & Bishwajit, 2020), or when analyzed physical activity domains, they did not report what would be the types (modalities) of physical activities practiced (Sperlich et al., 2020). The results found are relevant, as they strengthen the importance of regular physical activity in the positive health perception. However, it is also important to investigate whether specific physical activities, such as walking, have the same directions as studies mentioned above, aiming to provide subsidies for government agencies to establish public policies to promote assertive physical activity.

Thus, the present study aims to verify the association between weekly leisure walking time and positive self-rated health in the Brazilian adult and older adult population.

## Methods

### General aspects

This study used information collected in 2019 and is part of a project coordinated by the Ministry of Health of Brazil entitled “Surveillance System of Risk and Protection Factors of Noncommunicable Disease by Telephone Survey - VIGITEL” (Malta et al., 2015; Bernal et al., 2017; Brasil, 2020). This system aims to monitor health indicators of the Brazilian population aged ≥ 18 years through telephone survey and was carried out in 26 Brazilian capitals and the Federal District (Malta et al., 2015; Bernal et al., 2017; Brasil, 2020). Through this system, health promotion policies in Brazil are constantly restructured and evaluated.

Verbal consent by telephone was obtained before the questionnaire, free and informed consent was obtained from all participants at the time of data collection. The VIGITEL study in Brazil protocol was approved by National Ethics Committee on Research with Human Beings (CONEP/BRAZIL), and has been conducted in full accordance with ethical principles, including provisions of the World Medical Association Declaration of Helsinki (Ethical Application Ref: 65610017.1.0000.0008).

### Sampling

The sampling procedures used by the VIGITEL study aim to obtain, in each of the 26 state capitals and Federal District, probabilistic samples of the population of adults (≥ 18 years of age) residing in households served by at least one fixed telephone line. The system establishes minimum sample size of approximately 2 thousand individuals in each city to estimate, with 95% confidence coefficient and maximum error of two percentage points, the frequency of any risk factor in the adult population (Malta et al., 2015; Bernal et al., 2017; Brasil, 2020). Maximum errors of three percentage points are expected for specific estimates, according to sex, assuming similar proportions of men and women in the sample, as recommended by the World Health Organization—World Health Organization (1991). Smaller samples are accepted in locations where fixed telephone coverage is less than 40% of households and where the absolute number of households with a fixed telephone line is less than 50 thousand. In this case, estimates for the adult population will have maximum error of three percentage points, with the same error being four percentage points for gender-specific estimates (World Health Organization, 1991).

The first sampling stage consisted of drawing at least 5,000 telephone lines per city. This systematic and stratified by zip code draw was carried out based on the electronic registration of fixed telephone lines of telephone companies. Then, the lines drawn in each city are redrawn and divided into replicas of 200 lines, each replica reproducing the same proportion of lines per zip code of the original register. The division of the full sample into replicates is essentially due to the difficulty in estimating, in advance, the proportion of telephone lines in the register that will be eligible for the system (active residential lines) (Brasil, 2020). In 2019, from telephone registrations serving the 26 capitals and the Federal District, 197,600 telephone lines were initially drawn (on average 7,200 per city, distributed into 36 replicates of 200 lines each). In order to reach the minimum number of about 2 thousand interviews in each capital, an average of 36 replicas per city were used, ranging from 30 to 56 replicas (Brasil, 2020).

The second sampling stage consisted of the drawing of one of the adults (≥ 18 years of age) residing in the chosen household. This step is performed after the identification, among drawn lines, of those that are eligible for the system (Malta et al., 2015; Bernal et al., 2017; Brasil, 2020).

The lines that: (a) corresponded to companies; (b) no longer existed or were out of service; (c) did not answer to six call attempts made at different days and times, including Saturdays and Sundays and night periods were not eligible for the research. In 2019, calls were made to 197,600 telephone lines distributed in 988 replicas in the 26 capitals and the Federal District, identifying 75,789 eligible lines. At the end, 52,443 interviews were completed, which indicates system success rate of 69.2% (Brasil, 2020).

Of the total number of interviews carried out, this study analyzed information from the sample of people who reported practicing physical activity during leisure time, which was the primary aim of this article.

### Data collection procedure

Telephone interviews conducted in the survey were carried out between January and December 2019. The team responsible for the interviews—involving approximately 32 interviewers received prior training and was supervised during the system operation by technicians from the Department of Health Surveillance—Ministry of Health of Brazil.

The Consent Form was obtained orally because the research was carried out by telephone. The questions in the survey questionnaire covered the following indicators: (a) sociodemographic characteristics; (b) characteristics of the physical activity pattern; (c) self-reported body mass and height; (d) self-rated health status; (e) reference to previous medical hypertension and diabetes diagnosis; (f) use of medicines for chronic noncommunicable diseases.

The questionnaire construction process took into account several models of simplified questionnaires used by systems used for the monitoring of risk factors for chronic diseases (Remington et al., 1988; World Health Organization, 2001); the accumulated experience in system implantation tests carried out since 2003 in different Brazilian municipalities (Monteiro et al., 2005; Carvalhaes, Moura & Monteiro, 2008).

### Dependent variable

Self-rated health is the dependent variable in this study. This variable was collected by asking how the person would classify the state of his/her own health. Response options were “Very good”, “Good”, “Regular”, “Bad” and “Very bad”. For the analyses of the present study, this variable was dichotomized into positive self-rated health (categories “Very good” and “Good”) and negative self-rated health (categories “Regular”, “Bad” and “Very bad”). Several studies used the same classification (Barros et al., 2009; Szwarcwald et al., 2015; Habib et al., 2020; Haj-Younes et al., 2020). Self-rated health has shown adequate reproducibility and validity with health indicators in the Brazilian adult and elderly population (Theme Filha et al., 2015; Barros et al., 2009; Szwarcwald et al., 2015).

### Independent variable

The leisure walking time was the main exposure of this study. For this variable, walking on treadmill was not considered. This variable was collected using a reproducible and valid questionnaire for the Brazilian population (Monteiro et al., 2008; Del Duca et al., 2013; Moreira et al., 2017), which has been used in different studies developed in Brazil since 2006 (Florindo et al., 2009). Respondents were asked about the practice of physical activity during leisure in the last three months. The number of days per week that participants practiced walking and the time spent per day of this activity were computed through options of polytomous responses. The two questions (days/week and time/day) were combined to calculate the total time per week of leisure walking. Thus, the variable was categorized into: “<150 min/week”, “150–299 min/week” and “ ≥ 300 min/week”. This form of categorization was chosen because the current recommendations for physical activity reinforce the need for minimum of 150 min/week of moderate-intensity aerobic physical activity, as is the case with walking, and additional health benefits can be achieved with ≥ 300 min/week (Bull et al., 2020; World Health Organization, 2020).

### Adjustment variables

The literature shows that demographic, economic and factors related to the presence of chronic noncommunicable diseases influence the way people can assess their own health status (Szwarcwald et al., 2015; Cialani & Mortazavi, 2020; Haj-Younes et al., 2020). In this sense, in order to characterize the sample and adjust the multivariate analyses of the present research, the following variables were collected through questionnaire: sex (male/female); age in years (18–29 years; 30–39 years; 40–49 years; 50–59 years; 60–69 years; ≥ 70 years); schooling in years of study (≥ 12 years; 9–11 years; ≤ 8 years); skin color (white; brown; black; yellow; indigenous); marital status (single; married or stable relationship; divorced or widowed).

Food consumption was investigated using questionnaire built and validated for the Brazilian population (Sattamini, 2019) that is used in the VIGITEL system (Brasil, 2020). People were asked about the consumption of various foods the day before the interview. These foods were listed according to the Food Guide for the Brazilian Population (Brazil, 2014) and categorized into two large groups: (1) ultra-processed foods; (2) minimally ultra-processed or non-ultra-processed foods. Based on this information, each of the two food groups was categorized into consumption of five or more foods (Yes or No) on the day before the interview. The number of five or more foods was established because the VIGITEL system uses this cutoff point to monitor the Brazilian population (Brasil, 2020).

The practice of other types of physical activities during leisure time was investigated by validated questionnaire for the Brazilian population (Monteiro et al., 2008; Del Duca et al., 2013; Moreira et al., 2017). These practices were of moderate and vigorous intensity and, based on a list with different types of physical activity modalities, this variable was categorized as practice of “moderate” or “vigorous” intensity.

Additionally, people were asked about body mass (kg) and height (m). With this information, body mass index (BMI) was calculated to classify weight status into non-obesity (BMI <30 kg/m^2^) and obesity (BMI ≥ 30 kg/m^2^), as recommended in literature (World Health Organization, 2004).

For the presence of systemic arterial hypertension and diabetes mellitus, all respondents were asked about each disease specifically. The first question was whether any doctor had already diagnosed them with the disease. In addition, all respondents were asked about current medications they are using, and whether these medications are intended for systemic arterial hypertension and/or diabetes mellitus. If positive to any of the questions (diagnosis by a medical professional or use of medication), the participant was considered to have the disease. If all questions were negative, the participant was considered as not having the disease.

### Statistical analysis

Initially, all descriptive analyses were performed using absolute and relative frequency. In addition, mean and standard deviation for variable age was calculated.

For inferential statistics, the Chi-Square test of linear trend was used to test the association between leisure walking time and self-rated health status. Subsequently, crude binary logistic regression was used, which evaluated the association between the outcome of interest (positive self-rated health) and the other variables analyzed, with odds ratio (OR) estimates and 95% confidence intervals (95% CI).

The adjusted binary logistic regression model was constructed by including all variables analyzed, regardless of *p*-value of the crude analysis, remaining in the final model only variables associated with self-rated health at 5% significance level. In addition to OR and 95% CI estimates for adjusted associations, adjusted predicted probabilities of the final regression model were also estimated (Buis, 2007) using the ’marginal standardization’ method (Muller & MacLehose, 2014) in order to detect the average probabilities of positive self-rated health according to each of the categories related to leisure walking time.

In the binary logistic regression models, interactions between all covariates and the independent variable were tested; however, no interaction was significant and these interactions were not added to the final model. All analyses were conducted considering the sample weight of the study and the complex sample design. The Stata^®^ statistical software (StataCorp LLC, Texas, USA), version 15.0 was used in all analyses.

## Results

This study included 25,785 people aged ≥ 18 years (mean = 51.6; standard deviation = 18.0) from all states of Brazil who reported practicing walking as physical activity during leisure time. Most of the sample was female, aged ≥ 50 years, with ≥ 12 years of study, white skin color and in stable union or married. Of the sample, 86.7% reported moderate physical activity in the leisure time, 11% reported consuming five or more groups of ultra-processed food and 45.9% reported consuming five or more groups of non-ultra-processed food. Regarding chronic diseases, 17.3% of the sample had obesity, 31.4% had arterial hypertension, and 9.3% had diabetes mellitus. Regarding the practice of leisure walking, 40.2% of the sample reported walking for a period <150 min/week, 35.9% reported walking for a period from 150 to 299 min/week, and 23.9% reported walking for a period ≥ 300 min/week. Of the total respondents, 73.7% reported health as positive (Table 1).

**Figure 1:**
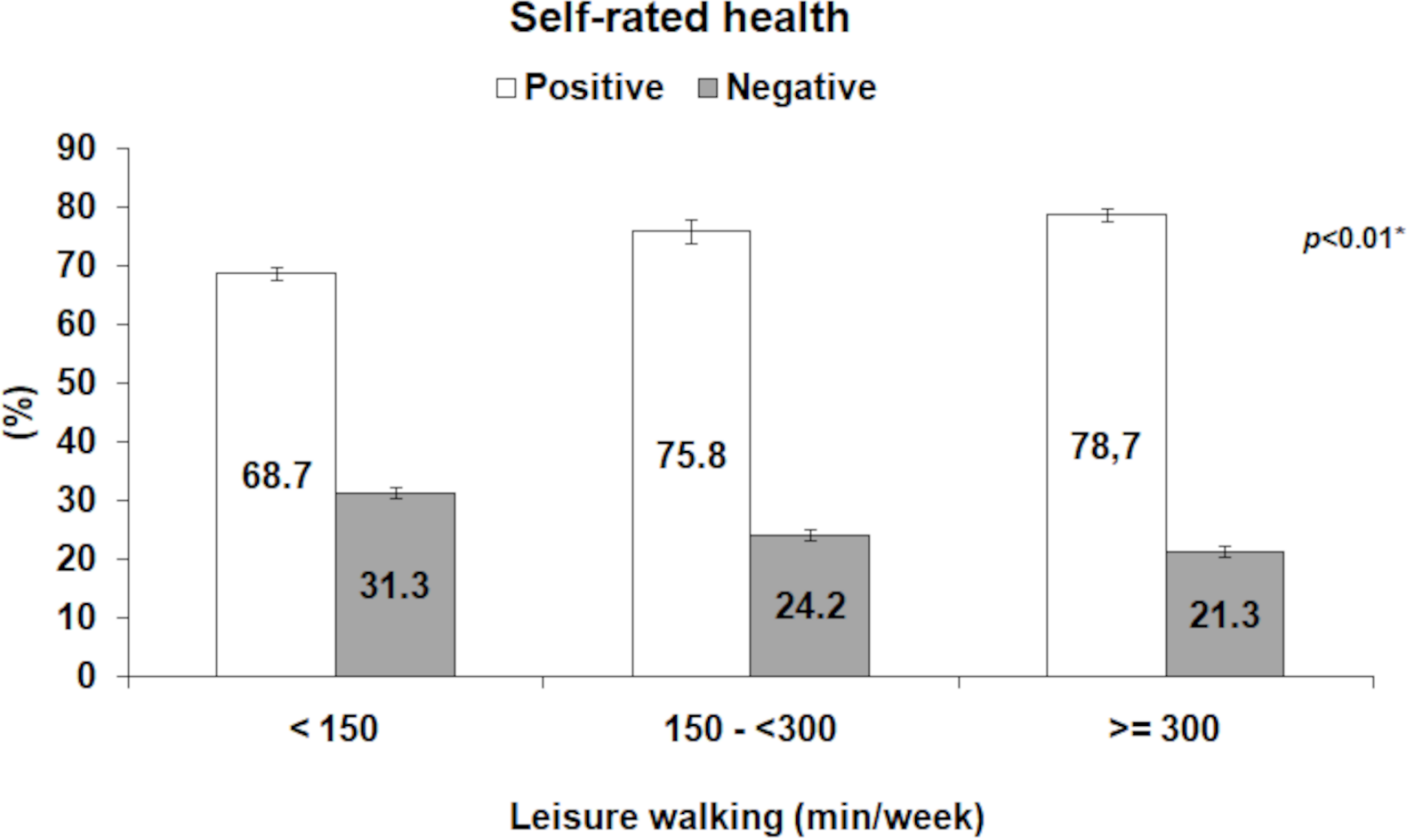
Prevalence of positive and negative self-rated health according to leisure walking time Brasil (2019).

The percentage of individuals who reported their health as positive increased as leisure walking time increased. On the other hand, the percentage of individuals who self-reported their health as negative decreased as leisure walking time increased (*p* < 0.01) (Fig. 1).

In the crude logistic regression model, it was found that individuals who reported leisure walking for a period from 150 to 299 minutes/week were 28% more likely of perceiving their health positively (OR = 1.28. 95% CI [1.11–1.47]) compared to those who reported walking for a period <150 minutes/week. In addition, individuals who reported walking for a period ≥ 300 minutes/week were 52% more likely of perceiving their health as positive (OR = 1.52. 95% CI [1.29–1.79]) than those who reported walking for a period <150 minutes/week. When adjusting the model for covariables, it was found that individuals who reported leisure walking for a period from 150 to 299 minutes/week and those who reported walking for a period ≥ 300 minutes/week were respectively 28% (OR = 1.28. 95% CI [1.10–1.48]) and 52% (OR = 1.52. 95% CI [1.20–1.59]) more likely of perceiving their health positively compared to those who reported walking for a period <150 minutes/week. Additionally, it was found that the population subgroups most likely of perceiving health in a positive way were male, people aged 18 to 69 years, those with more schooling, skin color white, people who practiced physical activity of vigorous intensity in leisure time, people who consume non-ultra-processed food and people without a diagnosis of obesity, hypertension and diabetes (Table 2).

**Figure 2:**
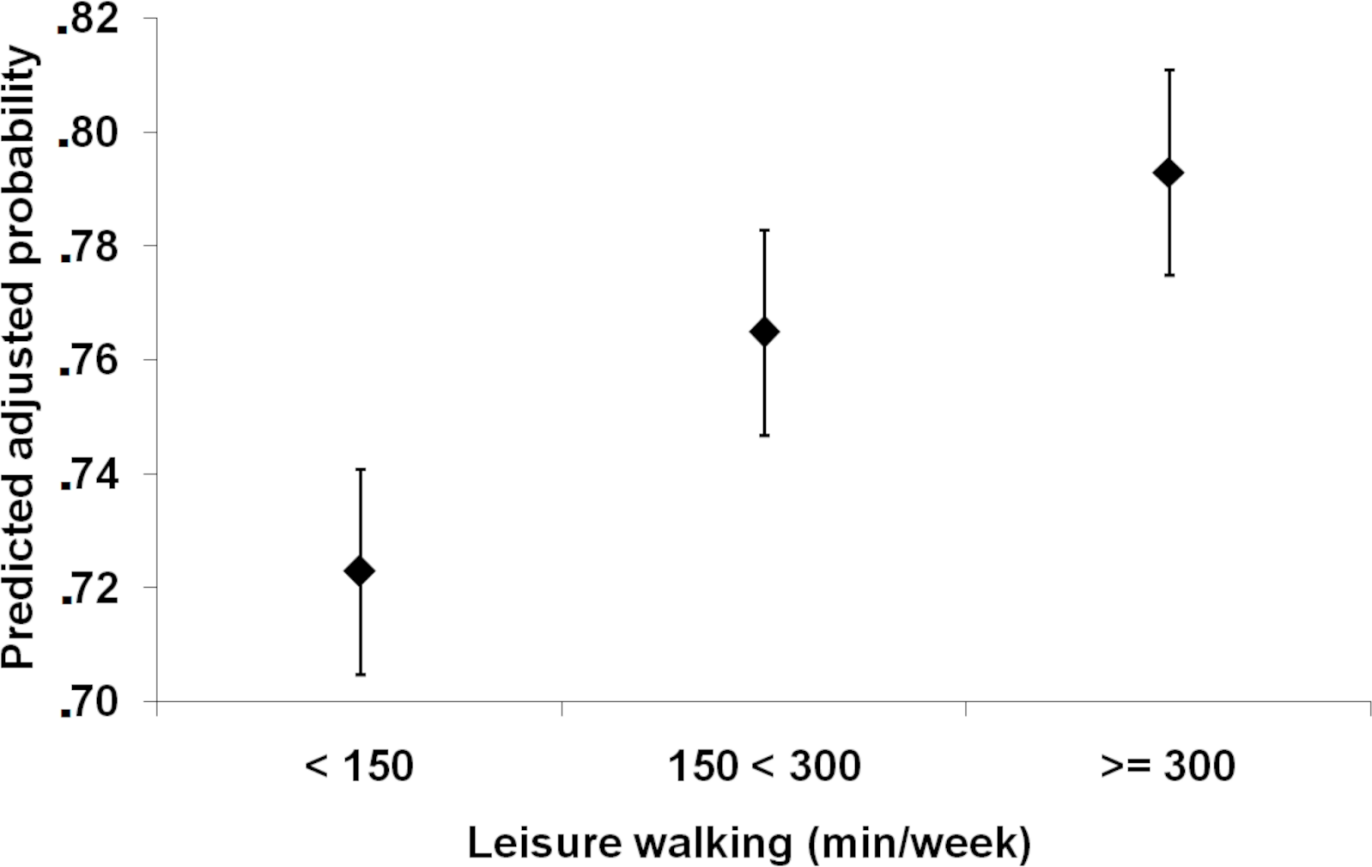
Adjusted predicted probability and 95% confidence intervals (95% CI) of positive self-rated health status according to leisure walking time in the Brazilian population.

From the adjusted logistic regression model, the predicted probability of the investigated population perceiving health in a positive way was estimated. Individuals who reported leisure walking time <150 minutes/week had 72.3% (95% CI [70.4–74.1]) probability of perceiving their health positively. Individuals who reported leisure walking time from 150 to 299 minutes/week had 76.6% (95% CI [75.0–78.3]) probability of perceiving their health positively. On the other hand, individuals who reported leisure walking time ≥ 300 minutes/week had 79.2% probability (95% CI [77.1–81.4]) of perceiving their health positively (Fig. 2).

## Discussion

The main result of the present study was the dose–response relationship between leisure walking and health perception in Brazilian adults, with time higher than 300 min/week of leisure walking representing higher probability of perceiving health positively and, this probability decreased in population subgroups that reported shorter leisure walking time. The results of the present study are in accordance with current evidence released by the World Health Organization (Bull et al., 2020; World Health Organization, 2020) on the benefits of physical activity practice for several health indicators, which health benefits are identified with the practice of aerobic physical activity of moderate intensity for minimum period of 150 min/week and additional benefits are found with greater volume of aerobic physical activity of moderate intensity.

**Table 1:** Descriptive characteristics of the investigated sample (Brasil, 2019).

	**n**	**%**	**95% CI**
**Sex**			
Male	10,314	40.0	(39.4–40.6)
Female	15,471	60.0	(59.4–60.5)
**Age group (years)**			
18–29	3,997	15.5	(15.0–15.9)
30–39	3,143	12.2	(11.7–12.6)
40–49	3,857	15.0	(14.5–15.3)
50–59	4,840	18.8	(18.2–19.3)
60–69	5,265	20.4	(19.9–20.9)
≥70	4,683	18.1	(17.6–18.6)
**Schooling**			
≤ 8 years	5,019	19.5	(18.9–19.9)
9–11 years	8,645	33.5	(32.9–34.1)
≥ 12 years	12,121	47.0	(46.3–47.6)
**Skin color**			
White	12,477	48.4	(47.7–48.9)
Brown	10,676	41.4	(40.8–42.0)
Black	2,078	8.0	(7.7–8.3)
Yellow	229	0.9	(0.7–1.1)
Indigenous	325	1.3	(1.1–1.4)
**Marital status**			
Single	8,287	32.1	(31.6–32.7)
Married/Stable union	12,855	49.9	(49.2–50.4)
Divorced/Widowed	4,643	18.0	(17.5–18.4)
**Physical activity intensity in leisure time**			
Moderate	22,367	86.7	(86.3–87.1)
Vigorous	3,418	13.3	(12.8–13.7)
**Consumption of ultra-processed food**			
No	22,961	89.0	(88.6–89.4)
Yes	2,824	11.0	(10.5–11.3)
**Consumption of non-ultra-processed food**			
No	13,949	54.1	(53.4–54.7)
Yes	11,836	45.9	(45.2–46.5)
**Obesity**			
No	21,313	82.7	(82.1–83.1)
Yes	4,472	17.3	(16.8–17.8)
**Hypertension**			
No	17,687	68.6	(68.0–69.1)
Yes	8,098	31.4	(30.8–31.9)
**Diabetes**			
No	23,389	90.7	(90.3–91.1)
Yes	2,396	9.3	(8.9–9.6)
**Self-rated health**			
Positive	18,991	73.7	(73.1–74.1)
Negative	6,794	26.3	(25.8–26.9)
**Leisure walking (minutes/week)**			
<150	10,375	40.2	(39.6–40.8)
≥150 <300	9,256	35.9	(35.3–36.4)
≥ 300	6,154	23.9	(23.3–24.4)

Health perception is considered a universal health marker and is closely related to the mental health of adults and older adults (Fayers & Sprangers, 2002; Burns et al., 2014). Thus, from results of the present study, it could be concluded that there was association between better mental health indicators and longer leisure walking time. Several physiological mechanisms can explain this interrelation, one of which is that the practice of physical activity stimulates the production of the endorphin neurohormone (Harber & Sutton, 1984), which is a substance related to the reduction of depressive and anxiety symptoms and improved self-esteem (Kubryak et al., 2012). However, what is not consensus in literature is the amount of physical activity that provides greater endorphin secretions and the physical activity threshold in which such endorphin secretion is ceased (Saanijoki et al., 2018). In this sense, this physiological mechanism is evidenced in literature; however, there is large field for further investigations.

**Table 2:** Logistic crude and adjusted regression analysis between self-rated health and other study variables (Brasil, 2019).

	**Positive self-rated health**			
	**Crude analysis**		**Adjusted analysis**	
	**OR (95% CI)**	***p***	OR (95% CI)^*^	***p***
**Leisure walking (minutes/week)**				
<150	1.00	<0.01	1.00	<0.01
≥150 <300	1.28 (1.11–1.47)		1.28 (1.10–1.48)	
≥ 300	1.52 (1.29–1.79)		1.52 (1.27–1.82)	
**Sex**				
Female	1.00	<0.01	1.00	<0.01
Male	1.31 (1.16–1.49)		1.38 (1.20–1.59)	
**Age group (years)**				
18–29	1.00	<0.01	1.00	<0.01
30–39	1.22 (0.99–1.50)		1.44 (1.14–1.81)	
40–49	0.98 (0.81–1.19)		1.32 (1.05–1.65)	
50–59	0.88 (0.73–1.06)		1.48 (1.18–1.87)	
60–69	0.73 (0.60–0.87)		1.46 (1.14–1.87)	
≥70	0.57 (0.47–0.68)		1.24 (0.94–1.62)	
**Schooling**				
≤ 8 years	1.00	<0.01	1.00	<0.01
9–11 years	1.32 (1.12–1.55)		1.10 (0.92–1.32)	
≥ 12 years	2.60 (2.20–3.08)		1.96 (1.61–2.36)	
**Skin color**				
White	1.00	<0.01	1.00	<0.01
Brown	0.68 (0.59–0.77)		0.74 (0.64–0.85)	
Black	0.65 (0.53–0.81)		0.74 (0.59–0.93)	
Yellow	0.88 (0.55–1.50)		0.80 (0.47–1.34)	
Indigenous	0.49 (0.32–0.76)		0.50 (0.32–0.81)	
**Marital status**				
Single	1.00	<0.01	1.00	0.14
Married/Stable union	0.90 (0.79–1.03)		1.13 (0.95–1.34)	
Divorced/Widowed	0.65 (0.54–0.78)		0.95 (0.75–1.20)	
**Physical activity intensity in leisure time**				
Moderate	1.00	<0.01	1.00	<0.01
Vigorous	1.44 (1.21–1.72)		1.36 (1.11–1.66)	
**Consumption of ultra-processed food**				
Yes	1.00	0.11	1.00	0.04
No	1.15 (0.96–1.37)		1.21 (1.01–1.47)	
**Consumption of non-ultra-processed food**				
No	1.00	<0.01	1.00	<0.01
Yes	1.52 (1.34–1.73)		1.44 (1.26–1.66)	
**Obesity**				
Yes	1.00	<0.01	1.00	<0.01
No	2.22 (1.91–2.57)		1.86 (1.57–2.20)	
**Hypertension**				
Yes	1.00	<0.01	1.00	<0.01
No	2.57 (2.25–2.93)		1.99 (1.68–2.35)	
**Diabetes**				
Yes	1.00	<0.01	1.00	<0.01
No	3.69 (2.98–4.55)		2.55 (2.03–3.19)	

**Notes.**

OR odds ratio CI confidence interval

* Adjusted analysis by leisure walking, sex, age, schooling, skin color, marital status, physical activity intensity in leisure time, consumption of ultra-processed food, consumption of non-ultra-processed food obesity, hypertension and diabetes.

Another physiological mechanism that can explain the interrelation between walking practice and possible improvement in mental health indicators is that higher levels of physical activity, such as walking, can change the mood state through increased production or positive regulation of brain monoamines norepinephrine, dopamine and serotonin (Lin & Kuo, 2013). However, most evidence on this interrelation comes from studies with animal models, mainly due to the difficulty in accurately measuring hormone levels after exercise in humans (Lin & Kuo, 2013). Although this hypothesis is well accepted for this explanation, further studies on the practice of a specific type of physical activity, such as walking, and brain monoamines are necessary, because the walking practice can reach greater number of practitioners compared to other types of physical activities.

The hypothesis called thermogenic, in which the practice of regular physical activity increases the internal body temperature, which, in turn, provides neurological changes associated with mood improvements even after the end of the exercise can also be explored (De Vries, 1981; Koltyn, 1997), which can result in improved health perception. This hypothesis still needs further investigation, however; it is a promising study field. In any case, all explanations previously given can strengthen the importance of leisure walking as one of the means of improving mental health indicators in adults and older adults.

Self-rated health is also related to the physical dimension of health, so that people with health problems that affect activities of the daily living, such as diabetics, obese and hypertensive patients, more frequently report negative assessment of their health status (Ge et al., 2019). One of the explanations for this is based on the sensations caused by these diseases, the use of controlled medications and some daily life limitations arising from these diseases (Ge et al., 2019). The present study found that Brazilian adults and older adults diagnosed with diseases were more likely of having negative self-rated health. This demonstrates that finding strategies for the prevention of non-communicable disease should be prioritized for the promotion of health in populations. One of the ways to prevent these diseases is through the regular practice of physical activities.

The present study has strengths such as the sample representativeness that included adults and older adults from all Brazilian geographic regions, with different socioeconomic characteristics. In addition, another strength is the analysis of the dose–response relationship between leisure walking and health perception. Such analysis reinforces recent recommendations on the health benefits of the practice of aerobic physical activity, such as walking.

The main limitations are the cross-sectional design, which does not allow establishing cause and effect relationships. In addition, the possibility of reverse causality is not ruled out. Another limitation of the present research is the fact that it did not investigate greater number of chronic noncommunicable diseases such as osteoporosis and depression, to be used as adjustment in statistical analyses, which are related to both outcome and the main exposure of this study. Finally, the investigation of walking time using a polytomous variable is another limitation, since information from a continuous measure could result in more detailed information about the interrelation between walking practice and health perception.

From this research, health professionals, especially Physical Education professionals, can prescribe the practice of walking as a strategy to improve the positive health perception of adults and older adults. Thus, through a modality that does not require sophisticated equipment for its practice, benefits to people’s health can be obtained. In Brazil, in particular, this modality can be prescribed in the Unified Health System (i.e., the Brazilian health care system) as a health-promoting strategy. Future studies should prioritize longitudinal designs so that the hypothesis of causality can be tested.

It could be concluded that longer leisure walking time was associated with positive self-rated health among adults and older adults in Brazil. In addition, leisure walking time longer than 300 min/week resulted in higher probability of positive health perception, regardless of sociodemographic factors, weight status, and diagnosed chronic diseases.

##  Supplemental Information

10.7717/peerj.11471/supp-1 Supplemental Information 1 Dataset

10.7717/peerj.11471/supp-2 Supplemental Information 2 Codebook - Dataset

10.7717/peerj.11471/supp-3 Supplemental Information 3 Questionnaire - English Language

10.7717/peerj.11471/supp-4 Supplemental Information 4 Original questionnaire - Portuguese language

## Additional Information and Declarations

### Data Availability

The following information was supplied regarding data availability: The raw measurements are available in the Supplementary File.
