# Supplementary material for: Longer leisure walking time is associated with positive self-rated health among adults and older adults: a Brazilian nationwide study
Source: PeerJ. 2021 May 17;9:e11471. doi: 10.7717/peerj.11471 (PMC8136276; doi:10.7717/peerj.11471)
Supplement: Supplemental Information 2 [file peerj-09-11471-s002.pdf]

Codebook - Database

Variable name In Portuguese	Variable name in English	storage type	display format	value label	Variable label in Portuguese	Variable label in English	Code	Label (in Portuguese)	Label (in English)
chave	Key	str11	%11s		chave	Key			
replica	Replica	byte	%8.0g		replica	Replica			
ano	Year	int	%8.0g		ano	Year			
mesfim	Month / end	str2	%2s		mes	Month			
cidade	City	byte	%8.0g	cidade	cidade	City	1	aracaju	aracaju
							2	belem	belem
							3	belo horizonte	belo horizonte
							4	boa vista	boa vista
							5	campo grande	campo grande
							6	cuiaba	cuiaba
							7	curitiba	curitiba
							8	florianopolis	florianopolis
							9	fortaleza	fortaleza
							10	goiania	goiania
							11	joao pessoa	joao pessoa
							12	macapa	macapa
							13	maceio	maceio
							14	manaus	manaus
							15	natal	natal
							16	palmas	palmas
							17	porto alegre	porto alegre
							18	porto velho	porto velho
							19	recife	recife
							20	rio branco	rio branco
							21	rio de janeiro	rio de janeiro
							22	salvador	salvador
							23	sao luis	sao luis
							24	sao paulo	sao paulo
							25	teresina	teresina
							26	vitoria	vitoria
							27	distrito federal	federal district
regiao	Region	byte	%8.0g	regiao	regiao	Region	1	centro-oeste	midwestern
							2	nordeste	northeastern

							3	norte	northern
							4	sudeste	southeastern
							5	sul	southern
bairro	Neighborhood	byte	%8.0g		bairro	Neighborhood			
data_ini	Date beg	str10	%10s		data inÍcio	Date beg			
hora_ini	Time beg	str8	%8s		hora inÍcio	Time beg			
data_fim	Date end	str10	%10s		data fim	Date end			
hora_fim	Time end	str8	%8s		hora fim	Time end			
duracao	Duration	str8	%8s		duração	Duration			
operador	Operator	str42	%42s		operador	Operator			
operadora	Operator	byte	%8.0g		operadora	Operator			
q6	q6	int	%8.0g		idade (anos)	Age (years)			
q7	q7	byte	%8.0g	q7	sexo		1	masculino	male
						Sex	2	feminino	female
							1	solteiro	single
							2	casado legalmente	legally married
civil	marital status	int	%8.0g	civil	estado conjugal atual	Current marital status	3	tem união estável há mais de seis meses	stable union for more than six months
							4	viúvo	widower
							5	separado ou divorciado	separated or divorced
							888	não quis informar	did not want to inform
							1	curso primário	elementary school
							2	admissão	admission
							3	curso ginasial ou ginásio	high school
							4	1º grau ou fundamental ou supletivo de 1º grau	elementary school or elementary school adult education
q8a		int	%8.0g	q8a	q8a-grau	q8a - grade	5	2º grau ou colégio ou técnico ou normal ou científico científico ou ensino médio ou supletivo de 2º grau	High school or technical or normal or scientific school or high school adult education
							6	3º grau ou curso superior	higher education
							7	pós-graduação (especialização, mestrado, doutorado)	Graduate studies (specialization, master's, doctorate)
							8	nunca estudou	illiterate
							777	não sabe	Do not know
							888	não quis responder	did not want to inform

q8b		byte	%8.0g		q8b-série (ano)	q8a – grade (year)			
q8_anos	q8_years	byte	%8.0g		anos de estudo	Years of study			
r128a		int	%8.0g	r128a	dirige		1	sim	yes
						driver	2	não	no
							888	não quis informar	did not want to answer
q9		float	%9.0g	LABB	peso (kg)		777	não sabe	Do not know
						Weight (kg)	888	não quis informar	did not want to answer
q11		int	%8.0g	LABB	altura (cm)		777	não sabe	Do not know
						Height (cm)	888	não quis informar	did not want to answer
q14		int	%8.0g	q14	gravidez		1	sim	yes
						Pregnancy	2	não	no
							777	não sabe	Do not know
q15		byte	%8.0g	LABC	feijão		1	1 a 2 dias por semana	1 to 2 days a week
							2	3 a 4 dias por semana	3 to 4 days a week
							3	5 a 6 dias por semana	5 to 6 days a week
						Beans	4	todos os dias (inclusive sábado e domingo)	everyday (including Saturday and Sunday)
							5	quase nunca	almost never
							6	nunca	never
q16		byte	%8.0g	LABC	freq horta		1	1 a 2 dias por semana	1 to 2 days a week
							2	3 a 4 dias por semana	3 to 4 days a week
							3	5 a 6 dias por semana	5 to 6 days a week
						Freq. vegetables	4	todos os dias (inclusive sábado e domingo)	everyday (including Saturday and Sunday)
							5	quase nunca	almost never
							6	nunca	never
q17		byte	%8.0g	LABC	freq horta crua		1	1 a 2 dias por semana	1 to 2 days a week
							2	3 a 4 dias por semana	3 to 4 days a week
							3	5 a 6 dias por semana	5 to 6 days a week
						Freq. raw vegetables	4	todos os dias (inclusive sábado e domingo)	everyday (including Saturday and Sunday)
							5	quase nunca	almost never
							6	nunca	never
q18		byte	%8.0g	LABD	quantas vezes		1	no almoço (1 vez no dia)	at lunch (once a day)
						How often	2	no jantar	at dinner
							3	no almoço e no jantar (2 vezes no dia)	at lunch and dinner (twice a day)
q19		byte	%8.0g	LABC	freq horta cozida	Freq. cooked	1	1 a 2 dias por semana	1 to 2 days a week

						vegetables			
							2	3 a 4 dias por semana	3 to 4 days a week
							3	5 a 6 dias por semana	5 to 6 days a week
							4	todos os dias (inclusive sábado e domingo)	everyday (including Saturday and Sunday)
							5	quase nunca	almost never
							6	nunca	never
q20		byte	%8.0g	LABD	quantas vezes		1	no almoço (1 vez no dia)	at lunch (once a day)
						How often	2	no jantar	at dinner
							3	no almoço e no jantar (2 vezes no dia)	at lunch and dinner (twice a day)
q21		byte	%8.0g	LABC	come carne		1	1 a 2 dias por semana	1 to 2 days a week
							2	3 a 4 dias por semana	3 to 4 days a week
							3	5 a 6 dias por semana	5 to 6 days a week
						Beef	4	todos os dias (inclusive sábado e domingo)	everyday (including Saturday and Sunday)
							5	quase nunca	almost never
							6	nunca	never
q22		byte	%8.0g	q22	gordura carne	Beef fat	1	tirar sempre o excesso de gordura	always remove excess fat
							2	comer com a gordura	eat with the fat
							3	não come carne vermelha com muita gordura	do not eat red meat with excess fat
q23		byte	%8.0g	LABC	come frango		1	1 a 2 dias por semana	1 to 2 days a week
							2	3 a 4 dias por semana	3 to 4 days a week
							3	5 a 6 dias por semana	5 to 6 days a week
						Chicken	4	todos os dias (inclusive sábado e domingo)	everyday (including Saturday and Sunday)
							5	quase nunca	almost never
							6	nunca	never
q24		byte	%8.0g	q24	pele de frango		1	tirar sempre a pele	always take the skin off
						Chicken skin	2	comer com a pele	eat with the skin
							3	não come pedaços de frango com pele	do not eat chicken pieces with skin
q25		byte	%8.0g	LABC	suco frutas		1	1 a 2 dias por semana	1 to 2 days a week
							2	3 a 4 dias por semana	3 to 4 days a week
							3	5 a 6 dias por semana	5 to 6 days a week
						Fruit juice	4	todos os dias (inclusive	everyday (including

								sábado e domingo)	Saturday and Sunday)
							5	quase nunca	almost never
							6	nunca	never
							1	1 copo	1 glass
q26		byte	%8.0g	q26	quanto suco	How much	2	2 copos	2 glasses
							3	3 ou mais copos	3 or more glasses
							1	1 a 2 dias por semana	1 to 2 days a week
							2	3 a 4 dias por semana	3 to 4 days a week
							3	5 a 6 dias por semana	5 to 6 days a week
q27		byte	%8.0g	LABC	freq fruta	Freq, fruits	4	todos os dias (inclusive sábado e domingo)	everyday (including Saturday and Sunday)
							5	quase nunca	almost never
							6	nunca	never
							1	1 vez no dia	Once a day
q28		byte	%8.0g	q28	quantas vezes	How often	2	2 vezes no dia	2 times a day
							3	3 ou mais vezes no dia	3 or more times a day
							1	1 a 2 dias por semana	1 to 2 days a week
							2	3 a 4 dias por semana	3 to 4 days a week
							3	5 a 6 dias por semana	5 to 6 days a week
q29		byte	%8.0g	LABC	freq refrigerante	Freq. soft drinks	4	todos os dias (inclusive sábado e domingo)	everyday (including Saturday and Sunday)
							5	quase nunca	almost never
							6	nunca	never
							1	normal	regular
q30		byte	%8.0g	q30	que tipo	What type	2	diet/ light/ zero	diet/ light/ zero
							3	ambos	both
							1	1 copo/latinha por dia	1 glass / can per day
							2	2 copos/latinhas por dia	2 glasses / cans per day
							3	3 copos/latinhas por dia	3 glasses / cans per day
q31		int	%8.0g	q31	qtdd refrigerante	Amount of soft drinks	4	4 copos/latinhas por dia	4 glasses / cans per day
							5	5 copos/latinhas por dia	5 glasses / cans per day
							6	6 ou mais copos/latinhas por dia	6 or more glasses / cans per day
							777	não sabe	Do not know
q32		byte	%8.0g	LABC	freqüência de leite		1	1 a 2 dias por semana	1 to 2 days a week

							2	3 a 4 dias por semana	3 to 4 days a week
							3	5 a 6 dias por semana	5 to 6 days a week
						Amount of milk	4	todos os dias (inclusive sábado e domingo)	everyday (including Saturday and Sunday)
							5	quase nunca	almost never
							6	nunca	never
q33		int	%8.0g	q33	tipo de leite		1	integral	whole
						Type of milk	2	desnatado ou semi-desnatado	skimmed or semi-skimmed
							3	os dois tipos	both types
							777	não sabe	Do not know
r143		byte	%8.0g	LABC	doces		1	1 a 2 dias por semana	1 to 2 days a week
							2	3 a 4 dias por semana	3 to 4 days a week
							3	5 a 6 dias por semana	5 to 6 days a week
						Sweets	4	todos os dias (inclusive sábado e domingo)	everyday (including Saturday and Sunday)
							5	quase nunca	almost never
							6	nunca	never
r146		byte	%8.0g	r146	quantas vezes come doce por dia	How many times eat sweets per day	1	1 vez no dia	Once a day
							2	2 vezes no dia	2 times a day
							3	3 ou mais vezes no dia	3 or more times a day
r144a		byte	%8.0g	LABC	troca almoço		1	1 a 2 dias por semana	1 to 2 days a week
							2	3 a 4 dias por semana	3 to 4 days a week
							3	5 a 6 dias por semana	5 to 6 days a week
						Exchanges lunch	4	todos os dias (inclusive sábado e domingo)	everyday (including Saturday and Sunday)
							5	quase nunca	almost never
							6	nunca	Never
r144b		byte	%8.0g	LABC	troca jantar		1	1 a 2 dias por semana	1 to 2 days a week
							2	3 a 4 dias por semana	3 to 4 days a week
							3	5 a 6 dias por semana	5 to 6 days a week
						Exchanges dinner	4	todos os dias (inclusive sábado e domingo)	everyday (including Saturday and Sunday)
							5	quase nunca	almost never
							6	nunca	never
q35		int	%8.0g	q35	bebida alcoólica	Alcoholic	1	sim	yes

						beverages			
							2	não	no
							888	não quis informar	did not want to inform
							1	1 a 2 dias por semana	1 to 2 days a week
						Freq. alcohol	2	3 a 4 dias por semana	3 to 4 days a week
							3	5 a 6 dias por semana	5 to 6 days a week
q36		byte	%8.0g	q36	freq alcool		4	todos os dias (inclusive sábado e domingo)	everyday (including Saturday and Sunday)
							5	menos de 1 dia por semana	less than 1 day a week
							6	menos de 1 dia por mês	less than 1 day per month
q37		byte	%8.0g	LABE	5 doses (homem)	5 doses (man)	1	sim	yes
							2	não	no
q38		byte	%8.0g	LABE	4 doses (mulher)	4 doses (woman)	1	sim	yes
							2	não	no
							1	em um único dia no mês	in a single day in the month
							2	em 2 dias	in 2 days
							3	em 3 dias	in 3 days
q39		int	%8.0g	q39	quantos dias	How often	4	em 4 dias	in 4 days
							5	em 5 dias	in 5 days
							6	em 6 dias	in 6 days
							7	em 7 ou mais dias	in 7 days or more
							777	não sabe	Do not know
r200		int	%8.0g	r200	num máximo de doses	Maximum number of doses			
							1	sim	yes
q40		int	%8.0g	q40	dirigiu após beber	Drove after drinking	2	não	no
							888	não quis informar	did not want to inform
							1	sempre	always
							2	algumas vezes	sometimes
q40b		int	%8.0g	q40b	independente da quantidade	Regardless of quantity	3	quase nunca	almost never
							4	nunca	never
							888	não quis informar	did not want to inform
q42		byte	%8.0g	LABE	exercício físico	Physical	1	sim	yes

						exercise			
							2	não	no
q43a		byte	%8.0g	q43a	qual	which	1	caminhada (não vale deslocamento para trabalho)	walking (not including commuting to work)
							2	caminhada em esteira	treadmill walking
							3	corrida (cooper)	running (cooper)
							4	corrida em esteira	treadmill running
							5	musculação	weightlifting
							6	ginástica aeróbica (spinning, step, jump)	aerobics (spinning, step, jump)
							7	hidroginástica	water aerobics
							8	ginástica em geral (alongamento, pilates, ioga)	general gymnastics (stretching, Pilates, yoga)
							9	natação	swimming
							10	artes marciais e luta (jiu-jitsu, karatê, judô, boxe, muay thai, capoeira)	martial arts and fighting (jiu-jitsu, karate, judo, boxing, muay thai, capoeira)
							11	bicicleta (inclui ergométrica)	bicycle (includes exercise bike)
							12	futebol/futsal	soccer / futsal
							13	basquetebol	basketball
							14	voleibol/futevolei	Volleyball / footvolley
							15	tênis	tennis
							16	dança (balé, dança de salão, dança do ventre)	dance (ballet, ballroom dance, belly dance)
							17	outros	others
q44		byte	%8.0g	LABE	pratica exercicio 1 vez na semana	Physical exercises once a week	1	sim	yes
							2	não	no
q45		byte	%8.0g	q45	freq exercicio	Freq. exercise	1	1 a 2 dias por semana	1 to 2 days a week
							2	3 a 4 dias por semana	3 to 4 days a week
							3	5 a 6 dias por semana	5 to 6 days a week
							4	todos os dias (inclusive sábado e domingo)	everyday (including Saturday and Sunday)
q46		byte	%8.0g	LABF	duração exercicio		1	menos que 10 minutos	less than 10 minutes
							2	entre 10 e 19 minutos	between 10 and 19

									minutes
							3	entre 20 e 29 minutos	between 20 and 29 minutes
						Exercise duration	4	entre 30 e 39 minutos	between 30 and 39 minutes
							5	entre 40 e 49 minutos	between 40 and 49 minutes
							6	entre 50 e 59 minutos	between 50 and 59 minutes
							7	60 minutos ou mais	60 minutes or more
q47		byte	%8.0g	LABE	trabalha	Works	1	sim	yes
							2	não	no
q48		int	%8.0g	LABG	anda bastante a pé	Walks much	1	sim	yes
							2	não	no
							777	não sabe	do not know
q49		int	%8.0g	LABG	carrega peso	Carries weight	1	sim	yes
							2	não	no
							777	não sabe	do not know
r147		int	%8.0g	r147	quantos dias faz essas atividades no trabalho	How many days performs these activities in work	555	menos de uma vez na semana	at least once a week
							888	não quis responder	did not want to inform
r148_hh		float	%9.0g	LABH	tempo de duração das atividades/ horas	Duration of activities / hours			
r148_mm		int	%8.0g	LABH	tempo de duração das atividades/ minutos	Duration of activities / minutes			
q50		byte	%8.0g	LABI	trajeto a pe ou bicicleta	Route on foot or bicycle	1	sim, todo o trajeto	yes, all the route
							2	sim, parte do trajeto	yes, part of the route
							3	não	no
q51		byte	%8.0g	LABF	duração do trajeto		1	menos que 10 minutos	less than 10 minutes
							2	entre 10 e 19 minutos	between 10 and 19 minutes
							3	entre 20 e 29 minutos	between 20 and 29 minutes
						Route duration	4	entre 30 e 39 minutos	between 30 and 39

									minutes
							5	entre 40 e 49 minutos	between 40 and 49 minutes
							6	entre 50 e 59 minutos	between 50 and 59 minutes
							7	60 minutos ou mais	60 minutes or more
q52		int	%8.0g	q52	curso/escola		1	sim	yes
						Course / school	2	não	no
							888	não quis informar	did not want to inform
q53		byte	%8.0g	LABI	como faz trajeto curso/escola	Course / school route	1	sim, todo o trajeto	yes, all the route
							2	sim, parte do trajeto	yes, part of the route
							3	não	
q54		byte	%8.0g	LABF	duração trajeto curso/escola		1	menos que 10 minutos	less than 10 minutes
							2	entre 10 e 19 minutos	between 10 and 19 minutes
						Course / school route duration	3	entre 20 e 29 minutos	between 20 and 29 minutes
							4	entre 30 e 39 minutos	between 30 and 39 minutes
							5	entre 40 e 49 minutos	between 40 and 49 minutes
							6	entre 50 e 59 minutos	between 50 and 59 minutes
							7	60 minutos ou mais	60 minutes or more
q55		byte	%8.0g	q55	faxina	House cleaning	1	eu sozinho	alone
							2	eu com outra pessoa	with someone else
							3	outra pessoa	another person
q56		byte	%8.0g	q56	parte pesada		1	o(a) sr(a)	you
						Heavy part	2	outra pessoa	another person
							3	ambos	both
r149		int	%8.0g	r149	quantos dias de faxina na semana	how many cleaning days in the week	555	menos de uma vez por semana	less than once a week

							888	não quis responder	did not want to answer
r150_hh		int	%8.0g	LABJ	tempo de faxina/ horas	cleaning time / hours			
r150_mm		int	%8.0g	LABJ	tempo de faxina/ minutos	cleaning time / minutes			
q59a		byte	%8.0g	q59a	quantas horas assiste tv / dia		1	menos de 1 hora	less than 1 hour
							2	entre 1 e 2 horas	between 1 and 2 hours
							3	entre 2 e 3 horas	between 2 and 3 hours
						how many hours of TV / day	4	entre 3 e 4 horas	between 3 and 4 hours
							5	entre 4 e 5 horas	between 4 and 5 hours
							6	entre 5 e 6 horas	between 5 and 6 hours
							7	mais de 6 horas	more than 6 hours
							8	não assiste televisão	do not watch television
q59b		int	%8.0g	q59b	uso de computador, tablet ou celular no tempo livre	use of computer, tablet or cell phone in free time	1	sim	yes
							2	não	no
							777	não sabe	do not know
q59c		byte	%8.0g	q59c	em média, este uso do computador, tablet ou celular		1	menos de 1 hora	less than 1 hour
						average time of computer, tablet or cell phone use	2	entre 1 e 2 horas	between 1 and 2 hours
							3	entre 2 e 3 horas	between 2 and 3 hours
							4	entre 3 e 4 horas	between 3 and 4 hours
							5	entre 4 e 5 horas	between 4 and 5 hours
							6	entre 5 e 6 horas	between 5 and 6 hours
							7	mais de 6 horas	more than 6 hours
q60		byte	%8.0g	q60	fumante		1	sim, diariamente	yes, daily
						Smoker	2	sim, mas não diariamente	yes, but not daily
							3	não	no
q61		int	%8.0g	LABK	cigarros diário	daily cigarettes			

q61a		int	%8.0g	LABK	cigarros semanal	weekly cigarettes			
							1	01 a 04	01 to 04
							2	05 a 09	05 to 09
							3	10 a 14	10 to 14
q61_fx		int	%8.0g	LABL	cigarros diário (agrupamento em faixas)	daily cigarettes (grouping in amounts)	4	15 a 19	15 to 19
							5	20 a 29	20 to 29
							6	30 a 39	30 to 39
							7	40 ou mais	40 or more
							888	não sabe/não quis responder	do not know / want to answer
							1	01 a 04	01 to 04
							2	05 a 09	05 to 09
							3	10 a 14	10 to 14
q61a_fx		int	%8.0g	LABL	cigarros semanal (agrupamento em faixas)	weekly cigarettes (grouping in amounts)	4	15 a 19	15 to 19
							5	20 a 29	20 to 29
							6	30 a 39	30 to 39
							7	40 ou mais	40 or more
							888	não sabe/não quis responder	Do not know / want to answer
q62		int	%8.0g	q62	idade inÍcio (anos)	start age (years)	777	não lembra	Do not remember
q63		byte	%8.0g	q63	tentou parar	tried to quit	1	sim	yes
							2	não	no
							1	sim, diariamente	yes, daily
q64		byte	%8.0g	q64	ex-fumante	ex-smoker	2	sim, mas não diariamente	yes, but not daily
							3	não	no
q67		int	%8.0g	q67	contato c/ fumo	contact with	1	sim	yes

					em casa	smoker at home			
							2	não	no
							888	não quis informar	did not want to inform
q68		int	%8.0g	q68	contato c/ fumo no trabalho	contact with smoker at work	1	sim	yes
							2	não	no
							888	não quis informar	did not want to inform
r157		int	%8.0g	r157	trabalha em local fechado		1	sim	yes
						works indoors	2	não	no
							888	não quis informar	did not want to inform
q69		int	%8.0g	q69	cor		1	branca	white
							2	preta	black
							3	amarela	yellow
						skin color	4	parda	brown
							5	indígena	indigenous
							777	não sabe	Do not know
							888	não quis informar	did not want to inform
q69_ou		str31	%31s		cor - outra	Color - other			
q70		byte	%8.0g	q70	outro fone		1	sim	yes
						another landline	2	não	no
q71		byte	%8.0g	q71	quantos		2	2 linhas	2 lines
						How many	3	3 linhas	3 lines
							4	4 linhas	4 lines
							5	5 linhas	5 lines
poor q74		int	%8.0g	q74	estado de saúde		1	muito bom	very good
							2	bom	good
						health status	3	regular	regular
							4	ruim	poor
							5	muito ruim	very poor
							777	não sabe	do not know
							888	não quis informar	did not want to inform
q75		int	%8.0g	LABM	pressão alta		1	sim	yes
						high blood pressure	2	não	no
							777	não lembra	do not remember

r203		int	%8.0g	r203	médico receitou medicamento pressão alta	doctor prescribed high blood pressure medicine	1	sim	yes
							2	não	no
							777	não lembra	do not remember
r129		int	%8.0g	r129	medicamento		1	sim	yes
						medicine	2	não	no
							777	não sabe	do not know
							888	não quis responder	did not want to inform
r130		int	%8.0g	LABN	onde consegue medicação pressão		1	unidade de saude do SUS	SUS health unit
						where gets medication to control high blood pressure	2	Farmácia Popular	Popular federal government pharmacy
							3	outro lugar	other place
							777	não sabe	do not know
							888	não quis responder	did not want to inform
r130a		int	%8.0g	LABN	onde consegue medicação pressão		1	unidade de saude do SUS	SUS health unit
						where gets medication to control high blood pressure	2	Farmácia Popular do Governo Federal	popular federal government pharmacy
							3	outro lugar (farmácia privada/particular, drogaria)	other place (private / private pharmacy, drugstore)
							777	não sabe	do not know
							888	não quis responder	did not want to inform
q76		int	%8.0g	LABM	diabetes		1	sim	yes
						diabetes	2	não	no
							777	não lembra	do not remember
r138		int	%8.0g	r138	diabetes apenas na gravidez		1	sim	yes
						diabetes only in pregnancy	2	não	no
							3	nunca engravidou	never got pregnant
							777	não lembra	do not remember

r202		int	%8.0g	r202	idade do ínicio do diabetes	age of onset of diabetes	777	não sabe / não lembra	do not know / remember
r204		int	%8.0g	r204	médico receitou medicamento diabetes	doctor prescribed diabetes medicine	1	sim	yes
							2	não	no
							777	não lembra	do not remember
r133a		int	%8.0g	LABO	comprimido diabetes		1	sim	yes
						diabetes tablets	2	não	no
							777	não sabe	do not know
							888	não quis responder	did not want to inform
r134c		int	%8.0g	LABN	onde consegue comprimido diabetes		1	unidade de saúde do sus	SUS health unit
							2	farmácia popular do governo federal	popular federal government pharmacy
						where do you get diabetes tablets	3	outro lugar (farmácia privada/particular, drogaria	other place (private / private pharmacy, drugstore)
							777	não sabe	Do not know
							888	não quis responder	did not want to inform
r133b		int	%8.0g	LABO	insulina diabetes		1	sim	yes
							2	não	no
						insulin diabetes	777	não sabe	Do not know
							888	não quis responder	did not want to inform
r134b		int	%8.0g	LABN	onde consegue insulina diabetes		1	unidade de saúde do sus	SUS health unit
							2	farmácia popular do governo federal	Popular federal government pharmacy
						where do you get insulin diabetes	3	outro lugar (farmácia privada/particular, drogaria	other place (private / private pharmacy, drugstore)
							777	não sabe	Do not know
							888	não quis responder	did not want to inform
q78		int	%8.0g	q78	colesterol ou triglicerides		1	sim	yes
						cholesterol or	2	não	no

						triglycerides			
							777	não sabe/não lembra	Do not know / remember
q79a		int	%8.0g	LABP	papanicolau		1	sim	yes
						pap smear	2	não	no
							777	não sabe	Do not know
q80		int	%8.0g	LABQ	tempo		1	menos de 1 ano	less than 1 year
							2	entre 1 e 2 anos	between 1 and 2 years
						time	3	entre 2 e 3 anos	between 2 and 3 years
							4	entre 3 e 5 anos	between 3 and 5 years
							5	5 anos ou mais	5 years or more
							777	não lembra	do not remember
q81		int	%8.0g	LABP	mamografia		1	sim	yes
						mammography	2	não	no
							777	não sabe	Do not knowv
q82		int	%8.0g	LABQ	tempo		1	menos de 1 ano	less than 1 year
							2	entre 1 e 2 anos	between 1 and 2 years
						time	3	entre 2 e 3 anos	between 2 and 3 years
							4	entre 3 e 5 anos	between 3 and 5 years
							5	5 ou mais anos	5 years or more
							777	não lembra	do not remember
q88		int	%8.0g	q88	posse de plano de saúde		1	sim, apenas 1	yes, only one
						health insurance	2	sim, mais de um	yes, more than one
							3	não	no
							888	não quis informar	did not want to inform
r135		int	%8.0g	LABR	nos últimos 12 meses foi multado?		1	sim	yes
						have you been fined in the past 12 months?	2	não	no
							777	não lembra	do not remember
							888	não quis responder	did not want to inform
r136		int	%8.0g	r136	qual o local		1	dentro da cidade (via urbana)	in the city (urban road)
							2	rodovia	highway
						what is the	3	ambos	both

						location			
							777	não lembra	do not remember
							888	não quis responder	did not want to inform
r153		int	%8.0g	LABR	passou em blitz		1	sim	yes
						passed through blitz	2	não	no
							777	não lembra	do not remember
							888	não quis responder	did not want to inform
r137a		int	%8.0g	LABR	parado em blitz		1	sim	yes
						stopped at blitz	2	não	no
							777	não lembra	do not remember
							888	não quis responder	did not want to inform
r154		int	%8.0g	LABR	convidado bafometro		1	sim	yes
						asked to perform breathalyzer test	2	não	no
							777	não lembra	do not remember
							888	não quis responder	did not want to inform
r155		int	%8.0g	LABR	fez o bafometro		1	sim	yes
						Performed the breathalyzer test	2	não	no
							777	não lembra	do not remember
							888	não quis responder	did not want to inform
r156		int	%8.0g	LABR	deu positivo		1	sim	yes
						was it positive?	2	não	no
							777	não lembra	do not remember
							888	não quis responder	
r900		int	%8.0g	r900	recebe bolsa família		1	sim	yes
						receives family allowance	2	não	no
							777	não sabe	do not know
obs_r900		str181	%181s		(observações) recebe bolsa família	(remarks) receives family allowance			

d1		int	%8.0g	d1	nos últimos 30 dias, ficou sem a insulina		1	sim	yes
						in the last 30 days, you have been without insulin	2	não	no
							777	não sabe	do not know
							888	não quis responder	did not want to inform
d2		int	%8.0g	d2	por que ficou sem a insulina		1	não tinha/estava em falta no posto de saúde/unidade de saúde/secretaria de saúde/sus	did not have / missing at the health unit / health secretariat / SUS
						why did you run out of insulin	2	porque não tinha dinheiro para comprar	had no money to buy
							3	esqueceu/não quis tomar/comprar/buscar	forgot / did not want to take / buy
							4	outro motivo	another reason
							777	não sabe	do not know
							888	não quis responder	did not want to inform
d3		int	%8.0g	d3	nos últimos 30 dias, ficou sem os comprimidos para controlar o diabetes		1	sim	yes
						in the past 30 days, you have been without medicines to control diabetes?	2	não	no
							777	não sabe	do not know
							888	não quis responder	did not want to inform
d4		byte	%8.0g	d4	por que ficou sem este medicamento (comprimido diabetes)	why did you run out of this medicine (diabetes tablets)	1	não tinha/estava em falta no posto de saúde/unidade de saúde/secretaria de saúde/sus	did not have / missing at the health unit / health secretariat / SUS
							2	porque não tinha dinheiro para comprar	had no money to buy
							3	esqueceu/não quis tomar/comprar/buscar	forgot / did not want to take / buy
							4	outro motivo	another reason
							777	não sabe	do not know
							888	não quis responder	did not want to inform

d5		int	%8.0g	d5	algum outro medicamento, deveria estar usando, nos últimos 30 dias, para a diabe		1	sim	yes
							2	não	no
							777	não sabe	do not know
						Is there any other medication that you should be using, in the last 30 days, for diabetes?	888	não quis responder	did not want to inform
d6		int	%8.0g	d6	por que ficou sem este medicamento (outro medicamento diabetes)		1	não tinha/estava em falta no posto de saúde/unidade de saúde/secretaria de saúde/sus	did not have / missing at the health unit / health secretariat / SUS
						why you ran out of this medicine (another diabetes medicine)	2	porque não tinha dinheiro para comprar	had no money to buy
							3	esqueceu/não quis tomar/comprar/buscar	forgot / did not want to take / buy
							4	outro motivo	another reason
							777	não sabe	do not know
							888	não quis responder	did not want to inform
moradores	Adult	byte	%8.0g		moradores	residents			
adultos	residents	byte	%8.0g		adultos	adults			
obs		str244	%244s		observações	remarks			
pesorake		double	%14.2fc		pesorake	pesorake			
fet		float	%12.0g	FET	Faixa etária		1	18 a 24	18 to 24 years
							2	25 a 34 anos	25 to 34 years
						age group	3	35 a 44 anos	35 to 44 years
							4	45 a 54 anos	45 to 54 years
							5	55 a 64 anos	55 to 64 years
							6	65 anos e mais	65 years and over
cat_esc		float	%9.0g		Categorias (idade | sexo) para imputação da	Categories (age | sex) for schooling			

					escolaridade	imputation			
fesc		float	%11.0g	FESC	Faixa de escolaridade	Schooling range	1	0 a 8 anos	0 to 8 years
							2	9 a 11 anos	9 to 11 years
							3	12 anos e mais	12 years and over
fxesc		float	%14.0g	FXESC	Faixa de escolaridade (RAKE)	Schooling range (RAKE)	1	0 a 7,9 anos	0 to 7.9 years
							2	8 a 10,9 anos	8 to 10.9 years
							3	11 a 14,9 anos	11 to 14.9 years
							4	15 a 20 anos	15 to 20 years
q9_i		float	%9.0g	LABB	peso (kg) - valores imputados	weight (kg) - imputed values			
q11_i		int	%8.0g	LABB	altura (cm) - valores imputados	height (cm) - imputed values			
pinterno		float	%9.0g						
