# Supplementary material for: Longer leisure walking time is associated with positive self-rated health among adults and older adults: a Brazilian nationwide study
Source: PeerJ. 2021 May 17;9:e11471. doi: 10.7717/peerj.11471 (PMC8136276; doi:10.7717/peerj.11471)
Supplement: Supplemental Information 3 [file peerj-09-11471-s003.pdf]

VIGITEL

Ministry of Health - Health Surveillance Secretariat
Surveillance of Risk and Protection Factors for Chronic Noncommunicable Diseases by Telephone Interviews (Vigitel) - 2019
Hotline - 136

INTERVIEW
City: XX, confirm the city: ☐ yes ☐ no (thank and close; exclude from sample bank and agenda).

1. Replica XX number of residents XX number of adults XX

2. Good morning / afternoon / evening. My name is XXXX. I'm from the Ministry of Health, is your phone number XXXX?
☐ Yes ☐ No - Sorry, I called the wrong number

3. I would like to speak to NAME OF SELECTED INDIVIDUAL. Is he / she home?

☐ Yes
☐ No - What is the best day of the week and time to talk to Mr. Mrs. NAME OF SELECTED INDIVIDUAL?
☐ Residence to return. Thank you, we will call you back. Close it.

3.a Can I talk to him / her now?
☐ Yes
☐ No - What is the best day of the week and time to Mr. Mrs. NAME OF SELECTED INDIVIDUAL?
☐ Residence to return. Thank you, we will call you back. Close it.

4. Have you been informed about the assessment conducted by the Ministry of Health?
☐ Yes (skip to Q5)
☐ No - The Ministry of Health is evaluating the health conditions of the Brazilian population and your telephone number was selected to participate in the interview. The interview should take about 10 minutes. Your answers will be kept confidential and will be used together with the answers of the other interviewees to provide a scenario of the current health conditions of the Brazilian population. For your safety, this interview will be recorded. If you have any questions about the research, you can clarify it directly on the "Disque Saúde" of the Ministry of Health on number 136. Would you like to write down the phone now or at the end of the interview? We inform that this research is regulated by the National Commission for Ethics in Research for Human Beings (CONEP) of the Ministry of Health.

5. Can we start the interview?
□ Yes (skip to Q6)
□ No - What is the best day of the week and time for us to talk?
□ Residence to return. Thank you, we will call you back. Close it.

Q6. How old are you? (only ≥18 years and <150)_________ years

Q7. Sex:
1 ( ) Male (skip Q14)   2 ( ) Female (if > 50 years old, skip Q14)

Q8. What is your schooling?
8A   8B - What was the last grade (year) you completed?

1 □ Elementary school	□ 1	□ 2	□ 3	□ 4
2 □ Admission	□ 4			
3 □High school	□ 1	□ 2	□ 3	□ 4

4 □ Regular elementary school or elementary school adult education
□ 1 □ 2 □ 3 □ 4 □ 5 □ 6 □ 7 □ 8
5 □ High school or technical or normal or scientific school or high school adult education
□ 1 □ 2 □ 3 □
6 □ Higher education
□ 1 □ 2 □ 3 □ 4 □ 5 □ 6 □ 7 □ 8 or +
7 □ Graduate studies (specialization, master's, doctorate) □ 1 or +
8 □ Illiterate
777 □ Do not know (only Q6> 60)
888 □ Did not want to answer

Q9. Do you know your weight (even if it is an approximate value)? (only ≥ 30 kg and <300 kg)
_______kg   777 □ Do not know   888 □ Did not want to inform

Q11. Do you know your height? (only ≥ 1.20 m and < 2.20 m)
_____m_____ cm   777 □ Do not know   888 □ Did not want to inform

Q12. Do you remember your approximate weight around 20 years of age?
(Only for Q6> 20 years)
1 □ Yes   2 □ No (skip to Q14)

Q13. Which was? (only ≥ 30 kg and <300 kg)
________kg   888 □ Did not want to inform

Q14. Are you pregnant at the moment? (Only if q6 <50 & q7 = 2)
1 □ Yes   2 □ No   777 □ Do not know

R190. Do you have license to drive a car, motorcycle and / or other vehicle?
1 □ yes   2 □ No   888 □ Did not want to inform

R128a. Do you drive a car, motorcycle and / or other vehicle?
1 □ yes   2 □ No   888 □ Did not want to inform

Now I'm going to ask you some questions about your eating habits

Q15. On how many days of the week, do you usually eat beans?
1 ( ) 1 to 2 days a week
2 ( ) 3 to 4 days a week
3 ( ) 5 to 6 days a week
4 ( ) Everyday (**including Saturday and Sunday**)
5 ( ) Almost never
6 ( ) Never

Q16. On how many days of the week, you usually eat at least one type of vegetable or legume (lettuce, tomato, cabbage, carrot, chayote, eggplant, zucchini - not including potatoes, cassava and yam)?
1 ( ) 1 to 2 days a week
2 ( ) 3 to 4 days a week
3 ( ) 5 to 6 days a week
4 ( ) Everyday (**including Saturday and Sunday**)
5 ( ) Almost never (skip to Q25)
6 ( ) Never (skip to Q25)

Q17. On how many days of the week, do you usually eat lettuce and tomato salad or any other raw vegetable or legume?
1 ( ) 1 to 2 days a week
2 ( ) 3 to 4 days a week
3 ( ) 5 to 6 days a week
4 ( ) Everyday (**including Saturday and Sunday**)
5 ( ) Almost never (skip to Q19)
6 ( ) Never (skip to Q19)

Q18. On an ordinary day, what type of salad do you usually eat:
1 ( ) At lunch (once a day)
2 ( ) At dinner or
3 ( ) At lunch and dinner (twice a day)

Q19. On how many days of the week, do you eat COOKED vegetables or legumes with food or soup, such as cabbage, carrots, chayote, eggplant, zucchini, not including potatoes and cassava?
1 ( ) 1 to 2 days a week
2 ( ) 3 to 4 days a week
3 ( ) 5 to 6 days a week
4 ( ) Every day (**including Saturday and Sunday**)
5 ( ) Almost never (skip to Q25)
6 ( ) Never (skip to Q25)

Q20. On an ordinary day, do you eat cooked vegetables or vegetables?
1 ( ) At lunch (once a day)
2 ( ) At dinner or
3 ( ) At lunch and dinner (twice a day)

Q25. On how many days of the week, do you usually drink natural fruit juice?
1 ( ) 1 to 2 days a week
2 ( ) 3 to 4 days a week
3 ( ) 5 to 6 days a week
4 ( ) Everyday (**including Saturday and Sunday**)
5 ( ) Almost never (skip to Q27)
6 ( ) Never (skip to Q27)

Q26. On an ordinary day, how many glasses of natural fruit juice do you usually drink?
1 ( ) 1
2 ( ) 2
3 ( ) 3 or more

Q27. On how many days of the week do you usually eat fruits?
1 ( ) 1 to 2 days a week
2 ( ) 3 to 4 days a week
3 ( ) 5 to 6 days a week
4 ( ) Everyday (**including Saturday and Sunday**)
5 ( ) Almost never (skip to Q29)
6 ( ) Never (skip to Q29)

Q28. On an ordinary day, how many times do you eat fruits?
1 ( ) 1 time in the day
2 ( ) 2 times a day
3 ( ) 3 or more times a day

Q29. On how many days of the week do you usually drink soft drinks or artificial juice?
1 ( ) 1 to 2 days a week
2 ( ) 3 to 4 days a week
3 ( ) 5 to 6 days a week
4 ( ) Everyday (**including Saturday and Sunday**)
5 ( ) Almost never (skip to R301)
6 ( ) Never (skip to R301)

Q30. What type?
1 ( ) Regular
2 ( ) Diet / light / zero
3 ( ) Both

Q31. On an ordinary day, how many glasses / cans do you usually drink?
1 □ 1 2 □ 2 3 □ 3 4 □ 4 5 □ 5 6 □ 6 or + 777 □ Do not know

Now I will list some foods and I would like you to tell me if you have eaten any of them yesterday (from when you woke up to when you went to sleep)

R301. I will start with natural or basic foods.
a. Lettuce, cabbage, broccoli, watercress or spinach
1□ Yes 2□ No

b. Pumpkin, carrot, sweet potato or okra / caruru
1□ Yes 2 □ No

c. Papaya, mango, yellow melon or pequi
1□ Yes 2□ No

d. Tomato, cucumber, zucchini, eggplant, chayote or beetroot
1□ Yes 2□ No

e. Orange, banana, apple or pineapple
1□ Yes 2□ No

f. Rice, pasta, polenta, couscous or green corn
1□ Yes 2□ No

g. Beans, peas, lentils or chickpeas
1□ Yes 2□ No

h. Common potato, cassava or yam
1□ Yes 2□ No

i. Beef, pork, chicken or fish
1☐ Yes 2☐ No

j. Fried, boiled or scrambled egg
1☐ Yes 2☐ No

k. milk
1☐ Yes 2☐ No

l. Peanuts, cashews or Brazil nuts
1☐ Yes 2☐ No

R302. Now I will list processed foods or products.
a. Soft drinks
1☐ Yes 2☐ No

b. Fruit juice in box or can
1☐ Yes 2☐ No

c. Drink powder
1☐ Yes 2☐ No

d. Chocolate drink
1☐ Yes 2☐ No

e. Flavored yogurt
1☐ Yes 2☐ No

f. Packet snack (or chips) or cookie / cracker
1☐ Yes 2☐ No

g. Cookie / sweet cookie, stuffed cookie or package cookie
1☐ Yes 2☐ No

h. Chocolate, ice cream, jelly, flan or other industrialized dessert
1☐ Yes 2☐ No

i. Sausage, bologna or ham
1☐ Yes 2☐ No

j. Loaf, hot dog or hamburger bread
1☐ Yes 2☐ No

k. Mayonnaise, ketchup or mustard
1☐ Yes 2☐ No

l. Margarine
1□ Yes 2□ No

m. Instant noodles, packaged soup, frozen lasagna or other ready-to-eat frozen dish
1□ Yes 2□ No

Now, some questions on the consumption of alcoholic beverages

Q35. Do you usually drink alcohol?
1 □ Yes 2 □ no (skip to R128a) 888 □ did not want to inform (skip to R128a)

Q36. How often do you consume alcoholic beverages?
1 ( ) 1 to 2 days a week
2 ( ) 3 to 4 days a week
3 ( ) 5 to 6 days a week
4 ( ) Everyday (**including Saturday and Sunday**)
5 ( ) Less than 1 day a week
6 ( ) Less than 1 day per month (skips to R128a)

Q37. In the past 30 days, did you consume five or more doses of alcoholic drink on a single occasion? (five doses of alcoholic drink would be five cans of beer, five glasses of wine or five doses of cachaça, whiskey or any other distilled alcoholic beverage) (for men only)

1 □ Yes (skip to Q39) 2 □ No (skip to R128a)

Q38. In the past 30 days, did you consume four or more doses of alcohol on a single occasion? (four doses of alcoholic drink would be 4 cans of beer, 4 glasses of wine or 4 doses of cachaça, whiskey or any other distilled alcoholic drink) (for women only)
1 □ yes 2 □ no (skip to R128a)

Q39. On how many days of the month did this occur?
1 ( ) In 1 single day in the month
2 ( ) In 2 days
3 ( ) In 3 days
4 ( ) In 4 days
5 ( ) In 5 days
6 ( ) In 6 days
7 ( ) In 7 days or more
777 □ Do not know

R200. On the days of the month that this occurred, what was the maximum number of doses consumed on a single occasion? (Example: a dose of alcoholic drink would be a can of beer, a glass of wine or a dose of cachaça, whiskey or any other distilled alcoholic drink - register in full doses - do not read)
___ ___ 777 □ Do not know

R128a. Do you drive a car, motorcycle and / or other vehicle?
1 □ yes 2 □ no 888 □ did not want to inform

Q40. On this day (or any of these days), did you drive right after drinking?
1 □ yes 2 □ no 888 □ did not want to inform

Q40b. Regardless of quantity, do you usually drive after consuming alcohol? (only for drivers - R128a = 1)
1 ( ) Always
2 ( ) Sometimes
3 ( ) Almost never
4 ( ) Never
888 □ Did not want to inform

In the next questions, we will ask about your daily physical activities

Q42. In the past three months, did you practice any type of physical exercise or sport?
1 □ Yes 2 □ No (skip to Q47) (not including physiotherapy)

Q43a. What is the main type of physical exercise or sport that you practiced?

RECORD ONLY THE FIRST MENTIONED

1 □ Walking (not including commuting to work)
2 □ Treadmill walking
3 □ Running (cooper)
4 □ Treadmill running
5 □ Bodybuilding
6 □ Aerobics (spinning, step, jump)
7 □ Water aerobics
8 □ Gymnastics in general (stretching, Pilates, yoga)
9 □ Swimming
10 □ Martial arts and fighting (jiu-jitsu, karate, judo, boxing, muay thai, capoeira)
11 □ Bicycle (includes exercise bike)
12 □ Soccer / futsal
13 □ Basketball
14 □ Volleyball / footvolley

15 □ Tennis
16 □ Dance (ballet, ballroom, belly dance)
17 □ Others_______________________________

Q44. Do you exercise at least once a week?
1□ yes 2□ no (skip to Q47)

Q45. How many days per week do you practice physical exercise or sport?
1 □ 1 to 2 days a week
2 □ 3 to 4 days a week
3 □ 5 to 6 days a week
4 □ Everyday (**including Saturday and Sunday**)

Q46. On the day you practice physical exercise or sport, how long does this activity last?
1 □ Less than 10 minutes
2 □ Between 10 and 19 minutes
3 □ Between 20 and 29 minutes
4 □ Between 30 and 39 minutes
5 □ Between 40 and 49 minutes
6 □ Between 50 and 59 minutes
7 □ 60 minutes or more

Q47. In the past three months, did you work?
1 □ Yes 2 □ No (skip to Q52)

Q48. In your work, do you walk much?
1 □ yes 2 □ no 777 □ Do not know

Q49. In your work, do you carry weight or do another heavy activity?
1 □ Yes 2 □ No (skip to Q50) 777 □ Do not know (skip to Q50)

R147. In a typical week, on how many days do you do these activities at work?
Number of days__________ 555 □ Less than once a week
888 □ Did not want to answer

R148. When you perform these activities, how long do they usually last?
HH: MM ______________________

Q50. Do you go to or return from work on foot or by bicycle?
1 □ Yes, the whole route 2 □ Yes, part of the route
3 □ no (skip to Q52)

Q51. How much time do you spend traveling to go to work and home in this route (on foot or by bicycle)?
1 □ Less than 10 minutes
2 □ Between 10 and 19 minutes
3 □ Between 20 and 29 minutes
4 □ Between 30 and 39 minutes
5 □ Between 40 and 49 minutes
6 □ Between 50 and 59 minutes
7 □ 60 minutes or more

Q52. Are you currently attending a course / school or taking someone to a course / school?
1 □ Yes 2 □ No (skip to Q55) 888 □ Did not want to inform (skip to Q55)

Q53. To go to or return from this course or school, do you travel by foot or bicycle?
1 □ Yes, the whole route 2 □ Yes, part of the route
3 □ No (skip to Q55)

Q54. How much time do you spend traveling to go to work and go back home on this route (on foot or by bicycle)? _________
1 □ Less than 10 minutes
2 □ Between 10 and 19 minutes
3 □ Between 20 and 29 minutes
4 □ Between 30 and 39 minutes
5 □ Between 40 and 49 minutes
6 □ Between 50 and 59 minutes
7 □ 60 minutes or more

Q55. Who usually cleans your house?
1 □ Me, alone (skip to R149) 2 □ Me, with another person
3 □ Another person (skip to Q59a)

Q56. The heaviest part of the cleaning is:
1 □ Me 2 □ Another person (skip to Q59a) 3 □ Both

R149. In a normal week, how many days do you clean your house?
Number of days____________ 555 □ Less than once a week
888 □ Did not want to answer

R150. How long does the cleaning usually take?
HH: MM ______________________

Q59a. On average, how many hours a day do you usually watch television?
1 ( ) Less than 1 hour
2 ( ) Between 1 and 2 hours
3 ( ) Between 2 and 3 hours
4 ( ) Between 3 and 4 hours
5 ( ) Between 4 and 5 hours
6 ( ) Between 5 and 6 hours
7 ( ) More than 6 hours
8 Does not watch television

Q59b. In your SPARE TIME, do you use a computer, tablet or cell phone to participate in social networks like Facebook, to watch movies or to play games?
1 Yes   2 No (skip to Q60)   777 Do not know (skip to Q60)

Q59c. On average, how many hours of your free time (excluding work), do you use the computer, tablet or cell phone in one day?
1 ( ) Less than 1 hour
2 ( ) Between 1 and 2 hours
3 ( ) Between 2 and 3 hours
4 ( ) Between 3 and 4 hours
5 ( ) Between 4 and 5 hours
6 ( ) Between 5 and 6 hours
7 ( ) More than 6 hours

In the next questions, we will ask about the smoking habit.

Q60. Do you currently smoke?
1 ( ) Yes, daily (go to Q61)
2 ( ) Yes, but not daily (skip to Q61a)   3 ( ) No (skip to Q64)

Q61. How many cigarettes do you smoke per day?_________(go to Q62)
1 1-4
2 5-9
3 10-14
4 15-19
5 20-29
6 30-39
7 40 or +

Q61a. How many cigarettes do you smoke per week? (only if Q60 = 2)
1 □ 1-4
2 □ 5-9
3 □ 10-14
4 □ 15-19
5 □ 20-29
6 □ 30-39
7 □ 40 or +

Q62. How old were you when you started smoking regularly? (only ≥ 5 years and ≥ 6)
________ years 777 □ Do not remember

Q63. Have you tried to quit smoking?
1 □ Yes (skip to Q67) 2 □ No (skip to Q67)

Q64. In the past, have you smoked?
1 ( ) Yes, daily
2 ( ) Yes, but not daily
3 ( ) No
(Go to Q69 if he / she lives alone and does not work)
(Go to Q68 if he / she lives alone and works)

Q67. Do any of the people who live with you smoke indoors?
1 □ yes 2 □ no 888 □ Did not want to inform

Q68. Does any colleague at work smoke in the same environment where you work? (only for Q47 = 1)
1 □ Yes 2 □ No (skip to R401 if Q60 = 1 or Q60 = 2; IF Q60 = 3, go to Q69)
888 □ Did not want to inform (skip to R401 if Q60 = 1 or Q60 = 2; IF Q60 = 3, go to Q69)

R157. If so, do you work indoors?
1□ Yes 2□ No 888 □ Did not want to inform

R401. The last time you bought cigarettes for your own use, how many cigarettes did you buy? (Interviewer: record the quantity and, when necessary, record the details of the unit) (Answer if q60 = 1 or q60 = 2)

Unit	Qtt.	Details
a. Cigarettes	__|	
b. Packs	__|	__| (How many cigarettes were in each pack)
c. Packages	__|	|_ (How many packs were in each package)<br>&<br>__|_ (How many cigarettes were in each pack)
I do not buy cigarettes for own use (skip to Q69)		

R402. In total, how much did you pay for that purchase?
R $

R403. Do you use electronic devices with liquid nicotine or chopped tobacco leaf (electronic cigarette, electronic hookah, heated cigarette or other electronic device) **to smoke or vaporize?** (Do not consider marijuana)
<READ OPTIONS>
1 ( ) Yes, daily
2 ( ) Yes, less than daily
3 ( ) No, but I used it in the past
4 ( ) Never used

Q69. What is your skin color or race?
1 ( ) White
2 ( ) Black
3 ( ) Yellow
4 ( ) Brown
5 ( ) Indigenous
777 □ Do not know
888 □ Did not want to inform

CIVIL. What is your current marital status?
1 ( ) Single
2 ( ) Married legally
3 ( ) Stable union for more than six months
4 ( ) Widowed
5 ( ) Separated or divorced
888 ( ) Did not want to inform

Q70. In addition to this phone number, do you have another landline number in your home?
1 Yes 2 No (skip to Q74)

Q71. If so: How many in total?__________ phone numbers or lines

Now, we are reaching the end of the questionnaire and we would like to know about your health status.

Q74. How would you rate your health status?
1 ( ) Very good
2 ( ) Good
3 ( ) Regular
4 ( ) Poor
5 ( ) Very poor
777 Do not know
888 Did not want to inform

Q75. Has any doctor ever told you that you have high blood pressure?
1 Yes
2 No (skip to Q76)
777 Do not remember (skip to Q76)

R 203. Has any doctor ever prescribed you medication to control high blood pressure?
1 Yes 2 No 777 Do not remember

R129. Are you currently taking any medications to control high blood pressure?
1 Yes
2 No (skip to Q76)
777 Do not know (skip to Q76)
888 Did not want to answer (skip to Q76)

R130a. How do you get medication to control high blood pressure?
1 ( ) SUS health unit
2 ( ) Popular federal government pharmacy
3 ( ) Other place (private / private pharmacy, drugstore)
777 Do not know
888 Did not want to answer

R174. In the past 30 days, have you been without any of the medications to control high blood pressure for some time? (APPLY if R129 = 1)
1 Yes 2 No 777 Do not remember

R175 When was the last time you measured your blood pressure?
1 □ Less than 6 months
2 □ Between 6 months and 1 year
3 □ Between 1 and 2 years
4 □ Between 2 and 3 years
5 □ 3 years or more
6 □ Never
777 □ Do not know / remember

Q76. Has any doctor ever told you that you have diabetes?
1 □ Yes 2 □ No (skip to Q79) 777 □ Do not remember (skip to Q79) (if Q7 = 1, man go to Q88)

R138. (If woman) Did you have diabetes only when you were pregnant? (only for Q7 = 2)
1 ( ) Yes
2 ( ) No
3 ( ) I never been pregnant
777 □ Do not remember

R202. How old were you when the doctor said you had diabetes?
_______years
777 □ Do not know / remember

R 204. Has any doctor ever prescribed you diabetes medication?
1 □ Yes 2 □ No 777 □ Do not remember

R133a. Are you currently taking any medication to control diabetes?
1 □ Yes
2 □ No (go to R133b)
777 □ Do not know (go to R133b)
888 □ Did not want to answer (go to R133b)

R134c. How do you get medications to control diabetes?
1 ( ) SUS health unit
2 ( ) Popular federal government pharmacy
3 ( ) Other place (private / private pharmacy, drugstore)
777 □ Do not know
888 □ Did not want to answer

D3. In the past 30 days, have you been without medications to control diabetes for some time?
1 □ Yes
2 □ No
777 □ Do not know
888 □ Did not want to answer

R133b. Are you currently using insulin to control diabetes?
1 Yes
2 No (if woman - Q7 = 2, go to Q79; if man - Q7 = 1, go to Q88)
777 Do not know (if woman - Q7 = 2, go to Q79; If man - Q7 = 1, go to Q88)
888 Did not want to answer (if woman - Q7 = 2, go to Q79; If man - Q7 = 1, go to Q88)

R134b. How do you get insulin to control diabetes? (APPLY if R133b = 1)
1 ( ) SUS health unit
2 ( ) Popular federal government pharmacy
3 ( ) Other place (private / private pharmacy, drugstore)
777 Do not know
888 Did not want to answer

D1. In the past 30 days, have you been without insulin for some time?
1 Yes
2 No
777 Do not know
888 Did not want to answer

R176. When was the last time you took a blood test to measure blood glucose, that is, blood sugar?
1 Less than 6 months
2 Between 6 months and 1 year
3 Between 1 and 2 years
4 Between 2 and 3 years
5 3 years or more 6 Never done
777 Do not know / remember

Q79a. Have you ever performed a Pap smear, a cervical cancer preventive exam? (female only - Q7 = 2)
1 Yes 2 No (skip to Q81) 777 Do not know (skip to Q81)

Q80. How long ago did you perform a Pap smear?
1 Less than 1 year
2 Between 1 and 2 years
3 Between 2 and 3 years
4 Between 3 and 5 years
5 5 years or more
777 Do not remember

Q81. Have you ever had a mammogram, x-ray of the breasts? (female only)
1 Yes 2 No (skip to Q88) 777 Do not know (skip to Q88)

Q82. How long ago did you have a mammogram?
1 Less than 1 year
2 Between 1 and 2 years
3 Between 2 and 3 years
4 Between 3 and 5 years
5 5 or more years
777 Do not remember

Q88. Do you have health plan or medical insurance?
1 ( ) Yes, just one
2 ( ) Yes, more than one
3 ( ) No
888 Did not want to answer (If you don't drive 0_R128 ≠ 1, go to R153)

R135. In the past 12 months, have you been fined for driving too fast on the road? (Only for drivers - R128a = 1)
1 ( ) Yes
2 ( ) No (skip to R153)
777 Do not remember (skip to R153)
888 Did not want to answer (skip to R153)

R136. In what location did you get fined?
1 ( ) In the city (urban road)
2 ( ) Highway
3 ( ) Both
777 Do not remember
888 Did not want to answer

R153. In the last 12 months, did you go to a blitz in your city?
1 ( ) Yes (if not driver [R128a ≠ 1] go to R179) (If R153 ≠ 1 & if driver [R128a = 1] go to 178)
2 ( ) No (if not driver [R128a ≠ 1] go to R179) (If R153 ≠ 1 & if driver [R128a = 1] go to 178)
777 Do not remember (if not driver [R128a ≠ 1] go to R179) (If R153 ≠ 1 & if driver [R128a = 1] go to 178)
888 Did not want to answer (if not driver [R128a ≠ 1] go to R179) (If R153 ≠ 1 & if driver [R128a = 1] go to 178)

R137a. In the past twelve months, as a driver, have been stopped at some traffic blitz in your city? (only for drivers - R128a = 1)
1 ( ) Yes
2 ( ) No (go to R178)
777 Do not remember (go to R178)
888 Did not want to answer (go to R178)

R154. (If Yes for R137a) Were you invited to take a breathalyzer test?
1 ( ) Yes
2 ( ) No (go to R178)
777 ☐ Do not remember (go to R178)
888 ☐ Did not want to answer (go to R178)

R155. (If Yes for R154) Did you take the breathalyzer test?
1 ( ) Yes
2 ( ) No (go to R178)
777 ☐ Do not remember (go to R178)
888 ☐ Did not want to answer (go to R178)

R156. (If Yes for R155). Was the breathalyzer test positive?
1 ( ) Yes
2 ( ) No
777 ☐ Do not remember
888 ☐ Did not want to answer

R178. In the past 30 days, did you use cell phone (calls, text messages, etc.) while driving a vehicle? (Only for drivers - R128a = 1)
1 ( ) Yes
2 ( ) No
777 ☐ Do not remember
888 ☐ Did not want to answer

R179. Do you or any other adult (> 18 years old) in your household have a cell phone?
1 ( ) Yes
2 ( ) No (go to R900)
777 ☐ Do not remember (go to R900)
888 ☐ Did not want to answer (go to R900)

R180. (If yes) Of <NUMBER OF ADULTS> adults in your household, how many have a cell phone?

____
777 ☐ Do not know
888 ☐ Did not want to answer

R900. Do you or someone in your family who lives with you receive family allowance?
1 ( ) Yes
2 ( ) No
777 ☐ Do not know

R901. Which person in your family who lives with you receives family allowance?

1 □ Interviewee himself
2 □ Spouse or partner
3 □ Child
4 □ Stepchild
5 □ Son-in-law or daughter-in-law
6 □ Father, mother, stepfather or stepmother
7 □ Father-in-law
8 □ Grandson / great-grandson
9 □ Brother
10 □ Grandfather or grandmother
11 □ Another relative - Which one? __________<register another relative>
777 □ Do not know
888 □ Did not want to answer

R902. How long has this person received the benefit?

1 □ Less than 2 years
2 □ Between 2 and 4 years
3 □ 5 years or more
777 □ Do not know

FOR ALL - FINAL CLOSING PAGE

Mr. XX, we thank you for your cooperation. If we have any questions we will call you again. If you did not write down the phone number at the beginning of the interview, please write down the “Disque-Saúde“ phone number.
If yes: The number is 136.
Observations (interviewer):

____
____
____

Note: Mention the answer alternatives to the interviewee only when they start with parentheses.
