# Supplementary material for: Longer leisure walking time is associated with positive self-rated health among adults and older adults: a Brazilian nationwide study
Source: PeerJ. 2021 May 17;9:e11471. doi: 10.7717/peerj.11471 (PMC8136276; doi:10.7717/peerj.11471)
Supplement: Supplemental Information 4 [file peerj-09-11471-s004.pdf]

VIGITEL

Ministério da Saúde – Secretaria de Vigilância em Saúde

Vigilância de Fatores de Risco e Proteção para Doenças Crônicas Não Transmissíveis por Entrevistas Telefônicas (Vigitel) – 2019

Disque-Saúde – 136

ENTREVISTA

Cidade: XX, confirma a cidade: ☐ sim ☐ não (agradeça e encerre; excluir do banco amostral e da agenda).

1. Réplica XX número de moradores XX número de adultos XX

2. Bom dia/tarde/noite. Meu nome é XXXX. Estou falando do Ministério da Saúde, o número do seu telefone é XXXX?

☐ Sim ☐ Não – Desculpe, liguei no número errado.

3. Sr.(a) gostaria de falar com o(a) Sr.(a) NOME DO SELECIONADO. Ele(a) está?

☐ Sim

☐ Não – Qual o melhor dia da semana e período para conversarmos com o(a) Sr.(a) NOME DO SELECIONADO?

☐ residência a retornar. Obrigado(a), retornaremos a ligação. Encerre.

3.a Posso falar com ele agora?

☐ Sim

☐ Não – Qual o melhor dia da semana e período para conversarmos com o(a) Sr.(a) NOME DO SELECIONADO?

☐ Residência a retornar. Obrigado(a), retornaremos a ligação. Encerre.

4. O(a) Sr.(a) foi informado sobre a avaliação que o Ministério da Saúde está fazendo?

☐ Sim (pule para Q5)

☐ Não – O Ministério da Saúde está avaliando as condições de saúde da população brasileira e o seu número de telefone e o(a) Sr.(a) foram selecionados para participar de uma entrevista. A entrevista deverá durar cerca de 10 minutos. Suas respostas serão mantidas em total sigilo e serão utilizadas junto com as respostas dos demais entrevistados para fornecer um retrato das condições atuais de saúde da população brasileira. Para sua segurança, esta entrevista será gravada. Caso tenha alguma dúvida sobre a pesquisa, poderá esclarecê-la diretamente no Disque Saúde do Ministério da Saúde, no telefone: 136. O(a) Sr.(a) gostaria de anotar o telefone agora ou no final da entrevista? Informamos que esta pesquisa está regulamentada pela Comissão Nacional de Ética em Pesquisa para Seres Humanos (CONEP) do Ministério da Saúde.

5. Podemos iniciar a entrevista?
☐ Sim (pule para Q6)
☐ Não – **Qual o melhor dia da semana e período para conversarmos?**
☐ Residência a retornar. Obrigado(a), retornaremos a ligação. Encerre.

Q6. Qual sua idade? (só aceita ≥18 anos e < 150) ____ anos

Q7. Sexo:
1( ) Masculino (pule a Q14) 2( ) Feminino (se > 50 anos, pule a Q14)

Q8. Até que série e grau o(a) Sr.(a) estudou?

8A	8B – Qual a última série (ano) o Sr.(a) completou?
1 ☐ Curso primário	☐ 1 ☐ 2 ☐ 3 ☐ 4
2 ☐ Admissão	☐ 4
3 ☐ Curso ginasial ou ginásio	☐ 1 ☐ 2 ☐ 3 ☐ 4

4 ☐ 1º grau ou fundamental ou supletivo de 1º grau
☐1 ☐2 ☐3 ☐4 ☐5 ☐6 ☐7 ☐8
5 ☐ 2º grau ou colégio ou técnico ou normal ou científico ou ensino médio ou supletivo de 2º grau
☐ 1 ☐ 2 ☐ 3 ☐
6 ☐ 3º grau ou curso superior
☐ 1 ☐ 2 ☐ 3 ☐ 4 ☐ 5 ☐ 6 ☐ 7 ☐ 8 ou +
7 ☐ Pós-graduação (especialização, mestrado, doutorado) ☐ 1 ou +
8 ☐ Nunca estudou
777 ☐ Não sabe (só aceita Q6 > 60)
888 ☐ Não quis responder

Q9. O(a) Sr.(a) sabe seu peso (mesmo que seja valor aproximado)? (só aceita ≥ 30 kg e < 300 kg)
_______ kg 777 ☐ Não sabe 888 ☐ Não quis informar

Q11. O(a) Sr.(a) sabe sua altura? (só aceita ≥ 1,20 m e < 2,20 m)
__ m ____ cm 777 ☐ Não sabe 888 ☐ Não quis informar

Q12. O(a) Sr.(a) lembra qual seu peso aproximado por volta dos 20 anos de idade?
(Apenas para Q6 > 20 anos)
1 ☐ Sim 2 ☐ Não (pule para a Q14)

Q13. Qual era? (Só aceitar ≥ 30 kg e < 300 kg)
_______ kg 888 ☐ Não quis informar

Q14. A Sra. está grávida no momento? (Só aceitar se q6 < 50 & q7 = 2)

1 ☐ Sim 2 ☐ Não 777 ☐ Não sabe

R190. O(a) Sr.(a) possui habilitação para dirigir carro, moto e/ou outro veículo?

1 ☐ Sim 2 ☐ Não 888 ☐ Não quis informar

R128a. O(a) Sr.(a) dirige carro, moto e/ou outro veículo?

1 ☐ Sim 2 ☐ Não 888 ☐ Não quis informar

Agora eu vou fazer algumas perguntas sobre sua alimentação

Q15. Em quantos dias da semana, o(a) Sr.(a) costuma comer feijão?

1 ( ) 1 a 2 dias por semana
2 ( ) 3 a 4 dias por semana
3 ( ) 5 a 6 dias por semana
4 ( ) Todos os dias (**inclusive sábado e domingo**)
5 ( ) Quase nunca
6 ( ) Nunca

Q16. Em quantos dias da semana, o(a) Sr.(a) costuma comer pelo menos um tipo de verdura ou legume (alface, tomate, couve, cenoura, chuchu, berinjela, abobrinha – não vale batata, mandioca ou inhame)?

1 ( ) 1 a 2 dias por semana
2 ( ) 3 a 4 dias por semana
3 ( ) 5 a 6 dias por semana
4 ( ) Todos os dias (**inclusive sábado e domingo**)
5 ( ) Quase nunca (pule para Q25)
6 ( ) Nunca (pule para Q25)

Q17. Em quantos dias da semana, o(a) Sr.(a) costuma comer salada de alface e tomate ou salada de qualquer outra verdura ou legume CRU?

1 ( ) 1 a 2 dias por semana
2 ( ) 3 a 4 dias por semana
3 ( ) 5 a 6 dias por semana
4 ( ) Todos os dias **(inclusive sábado e domingo)**
5 ( ) Quase nunca (pule para Q19)
6 ( ) Nunca (pule para Q19)

Q18. Num dia comum, o(a) Sr.(a) come este tipo de salada:

1 ( ) No almoço (1 vez ao dia)
2 ( ) No jantar ou
3 ( ) No almoço e no jantar (2 vezes ao dia)

Q19. Em quantos dias da semana, o(a) Sr.(a) costuma comer verdura ou legume COZIDO com a comida ou na sopa, como por exemplo, couve, cenoura, chuchu, berinjela, abobrinha, sem contar batata, mandioca ou inhame?

1 ( ) 1 a 2 dias por semana

2 ( ) 3 a 4 dias por semana

3 ( ) 5 a 6 dias por semana

4 ( ) Todos os dias **(inclusive sábado e domingo)**

5 ( ) Quase nunca (pule para Q25)

6 ( ) Nunca (pule para Q25)

Q20. Num dia comum, o(a) Sr.(a) come verdura ou legume cozido:

1 ( ) No almoço (1 vez ao dia)

2 ( ) No jantar ou

3 ( ) No almoço e no jantar (2 vezes ao dia)

Q25. Em quantos dias da semana o(a) Sr.(a) costuma tomar suco de frutas natural?

1 ( ) 1 a 2 dias por semana

2 ( ) 3 a 4 dias por semana

3 ( ) 5 a 6 dias por semana

4 ( ) Todos os dias **(inclusive sábado e domingo)**

5 ( ) Quase nunca (pule para Q27)

6 ( ) Nunca (pule para Q27)

Q26. Num dia comum, quantos copos o(a) Sr.(a) toma de suco de frutas natural?

1 ( ) 1

2 ( ) 2

3 ( ) 3 ou mais

Q27. Em quantos dias da semana o(a) Sr.(a) costuma comer frutas?

1 ( ) 1 a 2 dias por semana

2 ( ) 3 a 4 dias por semana

3 ( ) 5 a 6 dias por semana

4 ( ) Todos os dias **(inclusive sábado e domingo)**

5 ( ) Quase nunca (pule para Q29)

6 ( ) Nunca (pule para Q29)

Q28. Num dia comum, quantas vezes o(a) Sr.(a) come frutas?

1 ( ) 1 vez no dia

2 ( ) 2 vezes no dia

3 ( ) 3 ou mais vezes no dia

Q29. Em quantos dias da semana o(a) Sr.(a) costuma tomar refrigerante ou suco artificial?

1 ( ) 1 a 2 dias por semana

2 ( ) 3 a 4 dias por semana

3 ( ) 5 a 6 dias por semana

4 ( ) Todos os dias **(inclusive sábado e domingo)**

5 ( ) Quase nunca (pule para R301)

6 ( ) Nunca (pule para R301)

Q30. Que tipo?

1 ( ) Normal

2 ( ) *Diet/light/*zero

3 ( ) Ambos

Q31. Quantos copos/latinhas contuma tomar por dia?

1 ☐ 1 2 ☐ 2 3 ☐ 3 4 ☐ 4 5 ☐ 5 6 ☐ 6 ou + 777 ☐ Não sabe

Agora vou listar alguns alimentos e gostaria que o Sr.(a) me dissesse se comeu algum deles ontem (desde quando acordou até quando foi dormir)

R301. Vou começar com alimentos naturais ou básicos.

a. Alface, couve, brócolis, agrião ou espinafre
1☐ Sim 2☐ Não

b. Abóbora, cenoura, batata-doce ou quiabo/caruru
1☐ Sim 2o Não

c. Mamão, manga, melão amarelo ou pequi
1☐ Sim 2☐ Não

d. Tomate, pepino, abobrinha, berinjela, chuchu ou beterraba
1☐ Sim 2☐ Não

e. Laranja, banana, maçã ou abacaxi
1☐ Sim 2☐ Não

f. Arroz, macarrão, polenta, cuscuz ou milho verde
1☐ Sim 2☐ Não

g. Feijão, ervilha, lentilha ou grão de bico
1☐ Sim 2☐ Não

h. Batata comum, mandioca, cará ou inhame
1☐ Sim 2☐ Não

i. Carne de boi, porco, frango ou peixe
1☐ Sim 2☐ Não

j. Ovo frito, cozido ou mexido
1☐ Sim 2☐ Não

k. Leite
1☐ Sim 2☐ Não

l. Amendoim, castanha de caju ou castanha do Brasil/Pará
1☐ Sim 2☐ Não

R302. Agora vou relacionar alimentos ou produtos industrializados.

a. Refrigerante
1☐ Sim 2☐ Não

b. Suco de fruta em caixa, caixinha ou lata
1☐ Sim 2☐ Não

c. Refresco em pó
1☐ Sim 2☐ Não

d. Bebida achocolatada
1☐ Sim 2☐ Não
e. Iogurte com sabor
1☐ Sim 2☐ Não

f. Salgadinho de pacote (ou *chips*) ou biscoito/bolacha salgado
1☐ Sim 2☐ Não

g. Biscoito/bolacha doce, biscoito recheado ou bolinho de pacote
1☐ Sim 2☐ Não

h. Chocolate, sorvete, gelatina, *flan* ou outra sobremesa industrializada
1☐ Sim 2☐ Não

i. Salsicha, linguiça, mortadela ou presunto
1☐ Sim 2☐ Não

j. Pão de forma, de cachorro-quente ou de hambúrguer
1☐ Sim 2☐ Não

k. Maionese, *ketchup* ou mostarda
1☐ Sim 2☐ Não

l. Margarina
1☐ Sim 2☐ Não

m. Macarrão instantâneo, sopa de pacote, lasanha congelada ou outro prato pronto comprado congelado
1☐ Sim 2☐ Não

Agora, sobre o consumo de bebidas alcoólicas

Q35. O(a) Sr.(a) costuma consumir bebida alcoólica?

1 ☐ Sim 2 ☐ não (pula para R128a) 888 ☐ não quis informar (pula para R128a)

Q36. Com que frequência (a) Sr.(a) costuma consumir alguma bebida alcoólica?

1 ( ) 1 a 2 dias por semana
2 ( ) 3 a 4 dias por semana
3 ( ) 5 a 6 dias por semana
4 ( ) Todos os dias **(inclusive sábado e domingo)**
5 ( ) Menos de 1 dia por semana
6 ( ) Menos de 1 dia por mês (pula para R128a)

Q37. Nos últimos 30 dias, o Sr. chegou a consumir cinco ou mais doses de bebida alcoólica em uma única ocasião? (cinco doses de bebida alcoólica seriam cinco latas de cerveja, cinco taças de vinho ou cinco doses de cachaça, *whisky* ou qualquer outra bebida alcoólica destilada) (só para homens)

1 ☐ Sim (pule para Q39) 2 ☐ Não (pula para R128a)

Q38. Nos últimos 30 dias, a Sra. chegou a consumir quatro ou mais doses de bebida alcoólica em uma única ocasião? (4 doses de bebida alcoólica seriam 4 latas de cerveja, 4 taças de vinho ou 4 doses de cachaça, *whisky* ou qualquer outra bebida alcoólica destilada) (só para mulheres)

1 ☐ Sim 2 ☐ não (pula para R128a)

Q39. Em quantos dias do mês isto ocorreu?

1 ( ) Em 1 único dia no mês
2 ( ) Em 2 dias
3 ( ) Em 3 dias
4 ( ) Em 4 dias
5 ( ) Em 5 dias
6 ( ) Em 6 dias
7 ( ) Em 7 ou mais dias
777 ☐ Não sabe

R200. Nos dias do mês que isto ocorreu, qual foi o número máximo de doses consumido em uma única ocasião? (Exemplo: uma dose de bebida alcoólica seria uma lata de cerveja, uma taça de vinho ou uma dose de cachaça, *whisky* ou qualquer outra bebida alcoólica destilada – registrar em doses inteiras – não ler)

___ ___ 777 ☐ Não sabe

R128a. O(a) Sr.(a) dirige carro, moto e/ou outro veículo?

1 ☐ Sim 2 ☐ Não 888 ☐ Não quis informar

Q40. Neste dia (ou em algum destes dias), o(a) Sr.(a) dirigiu logo depois de beber?

1 ☐ Sim 2 ☐ Não 888 ☐ Não quis informar

Q40b. Independentemente da quantidade, o(a) Sr.(a) costuma dirigir depois de consumir bebida alcoólica? (apenas para quem dirige – R128a=1)

1 ( ) Sempre

2 ( ) Algumas vezes

3 ( ) Quase nunca

4 ( ) Nunca

888 ☐ Não quis informar

Nas próximas questões, vamos perguntar sobre suas atividades físicas do dia a dia

Q42. Nos últimos três meses, o(a) Sr.(a) praticou algum tipo de exercício físico ou esporte?

1 ☐ Sim 2 ☐ Não (pule para Q47) (não vale fisioterapia)

Q43a. Qual o tipo principal de exercício físico ou esporte que o(a) Sr.(a) praticou?

ANOTAR APENAS O PRIMEIRO CITADO

1 ☐ Caminhada (**não vale deslocamento para trabalho**)

2 ☐ Caminhada em esteira

3 ☐ Corrida (*cooper*)

4 ☐ Corrida em esteira

5 ☐ Musculação

6 ☐ Ginástica aeróbica (*spinning*, *step*, *jump*)

7 ☐ Hidroginástica

8 ☐ Ginástica em geral (alongamento, pilates, ioga)

9 ☐ Natação

10 ☐ Artes marciais e luta (*jiu-jítsu*, caratê, judô, boxe, *muay thai*, capoeira)

11 ☐ Bicicleta (inclui ergométrica)

12 ☐ Futebol/*futsal*

13 ☐ Basquetebol

14 ☐ Voleibol/futevôlei

15 ☐ Tênis

16 ☐ Dança (balé, dança de salão, dança do ventre)

17 ☐ Outros ________________________________

Q44. O(a) Sr.(a) pratica o exercício pelo menos uma vez por semana?

1☐ sim 2☐ não (pule para Q47)

Q45. Quantos dias por semana o(a) Sr.(a) costuma praticar exercício físico ou esporte?

1 ☐ 1 a 2 dias por semana

2 ☐ 3 a 4 dias por semana

3 ☐ 5 a 6 dias por semana

4 ☐ Todos os dias **(inclusive sábado e domingo)**

Q46. No dia que o(a) Sr.(a) pratica exercício ou esporte, quanto tempo dura esta atividade?

1 ☐ Menos de 10 minutos

2 ☐ Entre 10 e 19 minutos

3 ☐ Entre 20 e 29 minutos

4 ☐ Entre 30 e 39 minutos

5 ☐ Entre 40 e 49 minutos

6 ☐ Entre 50 e 59 minutos

7 ☐ 60 minutos ou mais

Q47. Nos últimos três meses, o(a) Sr.(a) trabalhou?

1 ☐ Sim 2 ☐ Não (pule para Q52)

Q48. No seu trabalho, o(a) Sr.(a) anda bastante a pé?

1 ☐ Sim 2 ☐ Não 777 ☐ não sabe

Q49. No seu trabalho, o(a) Sr.(a) carrega peso ou faz outra atividade pesada?

1 ☐ Sim 2 ☐ Não (pule para Q50) 777 ☐ Não sabe (pule para Q50)

R147. Em uma semana normal, em quantos dias o(a) Sr.(a) faz essas atividades no seu trabalho?

Número de dias ___ 555 ☐ Menos de 1 vez por semana 888 ☐ Não quis responder

R148. Quando realiza essas atividades, quanto tempo costuma durar?

HH:MM ________________________

Q50. Para ir ou voltar ao seu trabalho, faz algum trajeto a pé ou de bicicleta?

1 ☐ Sim, todo o trajeto 2 ☐ Sim, parte do trajeto 3 ☐ não (pule para Q52)

Q51. Quanto tempo o(a) Sr.(a) gasta para ir e voltar neste trajeto (a pé ou de bicicleta)?

1 ☐ Menos de 10 minutos

2 ☐ Entre 10 e 19 minutos

3 ☐ Entre 20 e 29 minutos

4 ☐ Entre 30 e 39 minutos

5 ☐ Entre 40 e 49 minutos

6 ☐ Entre 50 e 59 minutos

7 ☐ 60 minutos ou mais

Q52. Atualmente, o(a) Sr.(a) está frequentando algum curso/escola ou leva alguém em algum curso/escola?

1 ☐ Sim 2 ☐ Não (pule para Q55) 888 ☐ Não quis informar (pule para Q55)

Q53. Para ir ou voltar a este curso ou escola, faz algum trajeto a pé ou de bicicleta?

1 ☐ Sim, todo o trajeto 2 ☐ Sim, parte do trajeto 3 ☐ Não (pule para Q55)

Q54. Quanto tempo o(a) Sr.(a) gasta para ir e voltar neste trajeto (a pé ou de bicicleta)? _________

1 ☐ Menos de 10 minutos

2 ☐ Entre 10 e 19 minutos

3 ☐ Entre 20 e 29 minutos

4 ☐ Entre 30 e 39 minutos

5 ☐ Entre 40 e 49 minutos

6 ☐ Entre 50 e 59 minutos

7 ☐ 60 minutos ou mais

Q55. Quem costuma fazer a faxina da sua casa?

1 ☐ Eu, sozinho (pule para R149) 2 ☐ Eu, com outra pessoa 3 ☐ Outra pessoa (pule para Q59a)

Q56. A parte mais pesada da faxina fica com:

1 ( ) O(a) Sr.(a) ou 2 ( ) Outra pessoa (pule para Q59a) 3 ☐ Ambos

R149. Em uma semana normal, em quantos dias o(a) Sr.(a) realiza faxina da sua casa?

Número de dias ____ 555 ☐ Menos de 1 vez por semana 888 ☐ Não quis responder

R150. E quanto tempo costuma durar a faxina?

HH:MM ______________________

Q59a. Em média, quantas horas por dia o(a) Sr.(a) costuma ficar assistindo à televisão?

1 ( ) Menos de 1 hora

2 ( ) Entre 1 e 2 horas

3 ( ) Entre 2 e 3 horas

4 ( ) Entre 3 e 4 horas

5 ( ) Entre 4 e 5 horas

6 ( ) Entre 5 e 6 horas

7 ( ) Mais de 6 horas

8 ☐ Não assiste à televisão

Q59b. No seu TEMPO LIVRE, o Sr.(a) costuma usar computador, *tablet* ou celular para participar de redes sociais do tipo Facebook, para ver filmes ou para se distrair com jogos?

1 ☐ Sim 2 ☐ Não (pule para Q60) 777 ☐ Não sabe (pule para Q60)

Q59c. Em média, quantas horas do seu tempo livre (excluindo o trabalho), este uso do computador, *tablet* ou celular ocupa por dia?

1 ( ) Menos de 1 hora

2 ( ) Entre 1 e 2 horas

3 ( ) Entre 2 e 3 horas

4 ( ) Entre 3 e 4 horas
5 ( ) Entre 4 e 5 horas
6 ( ) Entre 5 e 6 horas
7 ( ) Mais de 6 horas

Nas próximas questões, vamos perguntar sobre o hábito de fumar

Q60. Atualmente, o(a) Sr.(a) fuma?
1 ( ) Sim, diariamente (ir para Q61)
2 ( ) Sim, mas não diariamente (pule para Q61a)
3 ( ) Não (pule para Q64)

Q61. Quantos cigarros o(a) Sr.(a) fuma por dia?________ (vá para Q62)
1 ☐ 1-4
2 ☐ 5-9
3 ☐ 10-14
4 ☐ 15-19
5 ☐ 20-29
6 ☐ 30-39
7 ☐ 40 ou +

Q61a. Quantos cigarros o(a) Sr.(a) fuma por semana? _________ (apenas se Q60=2)
1 ☐ 1-4
2 ☐ 5-9
3 ☐ 10-14
4 ☐ 15-19
5 ☐ 20-29
6 ☐ 30-39
7 ☐ 40 ou +

Q62. Que idade o(a) Sr.(a) tinha quando começou a fumar regularmente? (só aceita ≥ 5 anos e ≤ 6)
_______ anos 777 ☐ Não lembra

Q63. O(a) senhor(a) já tentou parar de fumar?
1 ☐ Sim (pule para Q67) 2 ☐ Não (pule para Q67)

Q64. No passado, o(a) Sr.(a) já fumou?
1 ( ) Sim, diariamente
2 ( ) Sim, mas não diariamente
3 ( ) Não
(Vá para Q69 se mora sozinho e não trabalha)
(Vá para Q68 se mora sozinho e trabalha)

Q67. Alguma das pessoas que moram com o(a) Sr.(a) costuma fumar dentro de casa?

1 ☐ Sim   2 ☐ Não   888 ☐ Não quis informar

Q68. Algum colega do trabalho costuma fumar no mesmo ambiente onde o(a) Sr.(a) trabalha? (só para Q47=1)

1 ☐ Sim   2 ☐ Não (pule para R401 se Q60 = 1 ou Q60 = 2; SE Q60 = 3, vá p/ Q69)
888 ☐ Não quis informar (pule para R401 se Q60 = 1 ou Q60 = 2; SE Q60 = 3, vá p/ Q69)

R157. Se sim, o(a) Sr.(a) trabalha em local fechado?

1☐ Sim   2☐ Não   888 ☐ Não quis informar

R401. A última vez em que o(a) Sr.(a) comprou cigarros para uso próprio, quantos cigarros comprou? (Entrevistador: registre a quantidade e, quando necessário, registre os detalhes da unidade) (Responder se q60 = 1 ou q60 = 2)

Unid.	Qtd.	Detalhes
a. Cigarros	__|__	
b. Maços (ou carteira)	__|__	__|__ (Quantos cigarros havia em cada maço)
c. Pacotes	__|__	__|__ (Quantos maços havia em cada pacote)<br>&<br>__|__ (Quantos cigarros havia em cada maço)
Não compro cigarros para uso próprio (pule para Q69)		

R402. No total, quanto o(a) Sr.(a) pagou por essa compra?

R$|__|__|__|__|__|__|__|__|__|__|__|

R403. O(a) Sr.(a) usa aparelhos eletrônicos com nicotina líquida ou folha de tabaco picado (cigarro eletrônico, narguilé eletrônico, cigarro aquecido ou outro dispositivo eletrônico) **para fumar ou vaporizar?** (Não considere o uso de maconha) **<LER OPÇÕES>**

1 ( ) Sim, diariamente

2 ( ) Sim, menos do que diariamente

3 ( ) Não, mas já usei no passado

4 ( ) Nunca usei

Q69. A sua cor ou raça é:

1 ( ) branca

2 ( ) Preta

3 ( ) Amarela

4 ( ) Parda

5 ( ) Indígena

777 ☐ Não sabe

888 ☐ Não quis informar

CIVIL. Qual seu estado conjugal atual?

1( ) Solteiro

2( ) Casado legalmente

3( ) Têm união estável há mais de seis meses

4( ) Viúvo

5( ) Separado ou divorciado

888( ) Não quis informar

Q70. Além deste número de telefone, tem outro número de telefone fixo em sua casa?

1 ☐ Sim 2 ☐ Não (pule para Q74)

Q71. Se sim: Quantos no total? ____números ou linhas telefônicas

Agora, estamos chegando ao final do questionário e gostaríamos de saber sobre seu estado de saúde.

Q74. O(a) Sr.(a) classificaria seu estado de saúde como:

1 ( ) Muito bom

2 ( ) Bom

3 ( ) Regular

4 ( ) Ruim

5 ( ) Muito ruim

777 ☐ Não sabe

888 ☐ Não quis informar

Q75. Algum médico já lhe disse que o(a) Sr.(a) tem pressão alta?

1 ☐ Sim

2 ☐ Não (pule para Q76)

777 ☐ Não lembra (pule para Q76)

R 203. Algum médico já lhe receitou algum medicamento para pressão alta?

1 ☐ Sim 2 ☐ Não 777 ☐ Não lembra

R129. Atualmente, o(a) Sr.(a) está tomando algum medicamento para controlar a pressão alta?

1 ☐ Sim

2 ☐ Não (pule para Q76)

777 ☐ Não sabe (pule para Q76)

888 ☐ Não quis responder (pule para Q76)

R130a. Como o(a) Sr.(a) consegue a medicação para controlar a pressão alta?

1 ( ) Unidade de saúde do SUS

2 ( ) Farmácia popular do governo federal

3 ( ) Outro lugar (farmácia privada/particular, drogaria)

777 ☐ Não sabe

888 ☐ Não quis responder

R174. Nos últimos 30 dias, o(a) sr(a). ficou sem algum dos medicamentos para controlar a pressão alta por algum tempo? (APLICAR se R129 = 1)

1 ☐ Sim 2 ☐ Não 777 ☐ Não lembra

R175 Quando foi a última vez que o(a) Sr.(a) mediu sua pressão arterial?

1 ☐ Menos de 6 meses

2 ☐ Entre 6 meses e 1 ano

3 ☐ Entre 1 e 2 anos

4 ☐ Entre 2 e 3 anos

5 ☐ 3 anos ou mais

6 ☐ Nunca

777 ☐ Não sabe / não lembra

Q76. Algum médico já lhe disse que o(a) Sr.(a) tem diabetes?

1 ☐ Sim 2 ☐ Não (pule para Q79) 777 ☐ Não lembra (pule para Q79)

(se Q7=1, homem vá para Q88)

R138. (Se mulher) O diabetes foi apenas quando estava grávida? (apenas para Q7=2)

1 ( ) Sim

2 ( ) Não

3 ( ) Nunca engravidou

777 ☐ Não lembra

R202. Que idade o(a) Sr.(a) tinha quando o médico disse que o(a) Sr.(a) tem diabetes?

_______ anos

777 ☐ Não sabe/não lembra

R 204. Algum médico já lhe receitou algum medicamento para diabetes?

1 ☐ Sim 2 ☐ Não 777 ☐ Não lembra

R133a. Atualmente, o(a) Sr.(a) está tomando algum comprimido para controlar o diabetes?

1 ☐ Sim

2 ☐ Não (vá para R133b)

777 ☐ Não sabe (vá para R133b)

888 ☐ Não quis responder (vá para R133b)

R134c. Como o(a) Sr.(a) consegue o comprimido para diabetes?

1 ( ) Unidade de saúde do SUS

2 ( ) Programa "Aqui tem Farmácia popular"

3 ( ) Outro lugar (farmácia privada/particular, drogaria)

777 ☐ Não sabe

888 ☐ Não quis responder

D3. Nos últimos 30 dias, o(a) Sr.(a) ficou sem algum dos comprimidos para controlar o diabetes por algum tempo?

1 ☐ Sim

2 ☐ Não

777 ☐ Não sabe

888 ☐ Não quis responder

R133b. Atualmente, o(a) Sr.(a) está usando insulina para controlar o diabetes?

1 ☐ Sim

2 ☐ Não (se mulher – Q7=2, vá para Q79 ; Se homem – Q7=1, vá para Q88)

777 ☐ Não sabe (se mulher – Q7=2, vá para Q79 ; Se homem – Q7=1, vá para Q88)

888 ☐ Não quis responder (se mulher – Q7=2, vá para Q79 ; Se homem – Q7=1, vá para Q88)

R134b. Como o(a) Sr.(a) consegue a insulina para diabetes? (APLICAR se R133b = 1)

1 ( ) Unidade de saúde do SUS

2 ( ) Programa "Aqui tem Farmácia popular"

3 ( ) Outro lugar (farmácia privada/particular, drogaria)

777 ☐ Não sabe

888 ☐ Não quis responder

D1. Nos últimos 30 dias, o(a) Sr.(a) ficou sem a insulina algum tempo?

1 ☐ Sim

2 ☐ Não

777 ☐ Não sabe

888 ☐ Não quis responder

R176. Quando foi a última vez que o(a) Sr.(a) fez exame de sangue para medir a glicemia, isto é, o açúcar no sangue?

1 ☐ Menos de 6 meses

2 ☐ Entre 6 meses e 1 ano

3 ☐ Entre 1 e 2 anos

4 ☐ Entre 2 e 3 anos

5 ☐ 3 anos ou mais

6 ☐ Nunca fez

777 ☐ Não sabe/não lembra

Q79a. A Sra. já fez alguma vez exame de Papanicolau, exame preventivo de câncer de colo do útero? (apenas para sexo feminino – Q7=2)

1 ☐ Sim 2 ☐ Não (pule para Q81) 777 ☐ Não sabe (pule para Q81)

Q80. Quanto tempo faz que a Sra. fez exame de Papanicolau?

1 ☐ Menos de 1 ano

2 ☐ Entre 1 e 2 anos

3 ☐ Entre 2 e 3 anos

4 ☐ Entre 3 e 5 anos

5 ☐ 5 anos ou mais

777 ☐ Não lembra

Q81. A Sra. já fez alguma vez mamografia, raio-X das mamas? (apenas para sexo feminino)

1 ☐ Sim 2 ☐ Não (pule para Q88) 777 ☐ Não sabe (pule para Q88)

Q82. Quanto tempo faz que a Sra. fez mamografia?

1 ☐ menos de 1 ano

2 ☐ entre 1 e 2 anos

3 ☐ entre 2 e 3 anos

4 ☐ entre 3 e 5 anos

5 ☐ 5 ou mais anos

777 ☐ Não lembra

Q88. O(a) Sr.(a) tem plano de saúde ou convênio médico?

1 ( ) Sim, apenas um

2 ( ) Sim, mais de um

3 ( ) Não

888 ☐ Não quis informar (Se não dirige 0_R128 ≠ 1, vá para R153)

R135. Nos últimos 12 meses, o Sr.(a) foi multado(a) por dirigir com excesso de velocidade na via? (Apenas para quem dirige – R128a = 1)

1 ( ) Sim

2 ( ) Não (pule para R153)

777 ☐ Não lembra (pule para R153)

888 ☐ Não quis responder (pule para R153)

R136. Qual o local que o(a) Sr.(a) foi multado?

1 ( ) Dentro da cidade (via urbana)

2 ( ) Rodovia

3 ( ) Ambos

777 ☐ Não lembra

888 ☐ Não quis responder

R153. Nos últimos 12 meses o(a) Sr.(a) você passou em uma *blitz* na sua cidade?

1 ( ) Sim (se não dirige [R128a ≠ 1] vá para R179) (Se R153 ≠ 1 & se dirige [R128a = 1] vá para 178)

2 ( ) Não (se não dirige [R128a ≠ 1] vá para R179) (Se R153 ≠ 1 & se dirige [R128a = 1] vá para 178)

777 ☐ Não lembra (se não dirige [R128a ≠ 1] vá para R179) (Se R153 ≠ 1 & se dirige [R128a = 1] vá para 178)

888 ☐ Não quis responder (se não dirige [R128a ≠ 1] vá para R179) (Se R153 ≠ 1 & se dirige [R128a = 1] vá para 178)

R137a. Nos últimos doze meses o Sr.(a), como condutor, foi parado em alguma *blitz* de transito na sua cidade? (apenas para quem dirige – R128a=1)

1 ( ) Sim

2 ( ) Não (vá para R178)

777 ☐ Não lembra (vá para R178)

888 ☐ Não quis responder (vá para R178)

R154. (Se Sim para R137a) E o(a) Sr.(a) foi convidado a fazer o teste de bafômetro?

1 ( ) Sim

2 ( ) Não (vá para R178)

777 ☐ Não lembra (vá para R178)

888 ☐ Não quis responder (vá para R178)

R155. (Se Sim para R154) E o(a) Sr.(a) fez o teste do bafômetro?

1 ( ) Sim

2 ( ) Não (vá para R178)

777 ☐ Não lembra (vá para R178)

888 ☐ Não quis responder (vá para R178)

R156. (Se Sim para R155). E o teste do bafômetro deu positivo?

1 ( ) Sim

2 ( ) Não

777 ☐ Não lembra

888 ☐ Não quis responder

R178. Nos últimos 30 dias, o(a) Sr.(a) fez uso de celular (ligações, mensagens de texto etc.) durante a condução de veículo? (Apenas para quem dirige – R128a = 1)

1 ( ) Sim

2 ( ) Não

777 ☐ Não lembra

888 ☐ Não quis responder

R179. O(a) Sr.(a) ou algum outro adulto (> 18 anos) de sua casa possui celular?

1 ( ) Sim

2 ( ) Não (vá para R900)

777 ☐ Não lembra (vá para R900)

888 ☐ Não quis responder (vá para R900)

R180. (Se sim) Dos <NÚMERO DE ADULTOS> adultos de sua casa, quantos possuem celular?

___ ___

777 ☐ Não sabe

888 ☐ Não quis responder

R900. Você ou alguém da sua família que more em sua casa recebe bolsa família?

1 ( ) Sim

2 ( ) Não

777 ☐ Não sabe

R901. Qual pessoa da sua família que mora na sua casa recebe o Bolsa Família?

1 ☐ Próprio entrevistado

2 ☐ Cônjuge ou companheiro(a)

3 ☐ Filho(a)

4 ☐ Enteado(a)

5 ☐ Genro ou nora

6 ☐ Pai, mãe, padrasto ou madrasta

7 ☐ Sogro(a)

8 ☐ Neto(a)/bisneto(a)

9 ☐ Irmão(a)

10 ☐ Avô ou avó

11 ☐ Outro parente – Qual? ____________________ <registrar outro parente>

777 ☐ Não sabe

888 ☐ Não quis responder

R902. Há quanto tempo essa pessoa recebe o benefício?

1 ☐ Menos de 2 anos

2 ☐ Entre 2 e 4 anos

3 ☐ 5 anos ou mais

777 ☐ Não sabe

PARA TODOS – PÁGINA FINAL DE ENCERRAMENTO

Sr.(a) XX Agradecemos pela sua colaboração. Se tivermos alguma dúvida voltaremos a lhe telefonar. Se não anotou o telefone no início da entrevista, gostaria de anotar o número de telefone do Disque-Saúde?

Se sim: O número é 136.

Observações (entrevistador):

____

____

____

Nota: Mencionar para o entrevistado as alternativas de resposta apenas quando as mesmas iniciarem por parênteses.
